# Elevated carbon dioxide levels lead to proteome-wide alterations for optimal growth of a fast-growing cyanobacterium, *Synechococcus elongatus* PCC 11801

**DOI:** 10.1038/s41598-019-42576-1

**Published:** 2019-04-18

**Authors:** Kanika Mehta, Damini Jaiswal, Monalisha Nayak, Charulata B. Prasannan, Pramod P. Wangikar, Sanjeeva Srivastava

**Affiliations:** 1 Department of Biosciences and Bioengineering, Indian Institute of Technology Bombay, Powai, Mumbai, 400076 India; 2 Department of Chemical Engineering, Indian Institute of Technology Bombay, Powai, Mumbai, 400076 India; 3 DBT-Pan IIT Center for Bioenergy, Indian Institute of Technology Bombay, Powai, Mumbai, 400076 India; 4 Wadhwani Research Center for Bioengineering, Indian Institute of Technology Bombay, Powai, Mumbai, 400076 India

**Keywords:** Proteome, Metabolic engineering

## Abstract

The environmental considerations attributing to the escalation of carbon dioxide emissions have raised alarmingly. Consequently, the concept of sequestration and biological conversion of CO_2_ by photosynthetic microorganisms is gaining enormous recognition. In this study, in an attempt to discern the synergistic CO_2_ tolerance mechanisms, metabolic responses to increasing CO_2_ concentrations were determined for *Synechococcus elongatus* PCC 11801, a fast-growing, novel freshwater strain, using quantitative proteomics. The protein expression data revealed that the organism responded to elevated CO_2_ by not only regulating the cellular transporters involved in carbon-nitrogen uptake and assimilation but also by inducing photosynthesis, carbon fixation and glycolysis. Several components of photosynthetic machinery like photosystem reaction centers, phycobilisomes, cytochromes, etc. showed a marked up-regulation with a concomitant downshift in proteins involved in photoprotection and redox maintenance. Additionally, enzymes belonging to the TCA cycle and oxidative pentose phosphate pathway exhibited a decline in their expression, further highlighting that the demand for reduced cofactors was fulfilled primarily through photosynthesis. The present study brings the first-ever comprehensive assessment of intricate molecular changes in this novel strain while shifting from carbon-limited to carbon-sufficient conditions and may pave the path for future host and pathway engineering for production of sustainable fuels through efficient CO_2_ capture.

## Introduction

**Figure 1:**
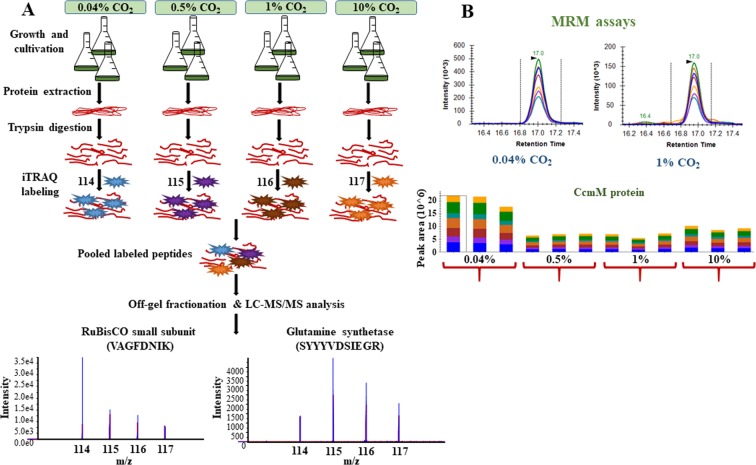
Quantitative proteomics study of *Synechococcus* 11801 grown at increasing CO_2_ levels. (**A**) Schematic illustration of the overall experimental strategy employed in the discovery-phase global quantitative proteomics study. (**B**) MRM assays to verify the iTRAQ results. A representative image of the MRM assay of CcmM protein has also been shown.

Cyanobacteria are gram-negative, oxygenic photoautotrophs that harness sunlight to fix inorganic carbon into biomass. With a rise in carbon dioxide levels and its predicted atmospheric concentration reaching approximately 1072 ppmv (parts per million by volume) by the year 2100^1^, the concept of CO_2_ uptake and mitigation by photosynthetic microorganisms has gained immense impetus^2^. Furthermore, to circumvent environmental concerns like diminishing fossil fuel reserves, their increasing costs and mounting CO_2_ emissions, the focus has recently shifted towards the development of alternative sustainable fuels with abated greenhouse release^2,3^. Cyanobacteria that typically capture solar energy to synthesize organic molecules are currently also being explored to produce diverse non-natural products using synthetic biology and metabolic engineering tools due to their pliable metabolism^4–8^. An ideal host cyanobacterium needs to be fast-growing, high light and CO_2_ tolerant, and genetically amenable^9^. Although pathway engineering approaches have been demonstrated to produce biochemicals/biofuels from the model cyanobacteria strains, their titers are not comparable to the heterotrophic model organisms like *E*. *coli*. In addition, the use of these model cyanobacteria strains in industrial settings poses certain limitations. For instance, the model organisms *Synechococcus elongatus* PCC 7942 and *Synechocystis* sp. PCC 6803 (hereafter referred to as *Synechococcus* 7942 and *Synechocystis* 6803, respectively) though naturally transformable, are slow-growing and cannot tolerate high light intensity. Other fast-growing strains like *Synechococcus* sp. PCC 7002 and *Synechococcus elongatus* UTEX 2973^10^ (hereafter referred to as *Synechococcus* 7002 and *Synechococcus* 2973, respectively) either require vitamin B_12_ as an additional supplement or are not naturally transformable. We have chosen here a recently reported strain from a local freshwater source, *Synechococcus elongatus* PCC 11801 (hereafter referred to as *Synechococcus* 11801), a close homolog of the model organism *Synechococcus* 7942 that possesses all the above-mentioned attributes which qualify it to be a future host strain^11^. The physiological characterization revealed that this local isolate is tolerant to high levels of CO_2_, light, temperature, salts and butanol. Additionally, since the strain grew without any supplementation and was found to be naturally transformable, it seems like a promising candidate for further characterization. Despite the high CO_2_ fixation potential of cyanobacteria, the underlying molecular modulations involved in the sequestration of CO_2_ are poorly understood. Since proteins are directly in control of various cellular functions, ascertaining protein abundances is likely to provide salient cues to the intracellular rearrangements occurring during environmental perturbations like high CO_2_ stress. Few studies have reported the proteomics alterations in cyanobacteria and microalgae grown under CO_2_ replete conditions, but their research was mainly focused either on the effects of high CO_2_ on CCM (CO_2_ concentrating mechanisms) or on extracellular proteins^12,13^, which highlights the paucity of detailed proteomics investigations at high CO_2_ conditions. Therefore, the present study was undertaken to understand the strategy adopted by *Synechococcus* 11801 in biological sequestration of CO_2_ using a high-throughput iTRAQ (isobaric tags for relative and absolute quantitation) based quantitative proteomics approach (Fig. 1A). Furthermore, some of the top hit proteins were validated using MRM (multiple reaction monitoring) assays (Fig. 1B). These proteins displayed a substantially altered expression and represented the major altered physiological pathways. A comprehensive proteomics study on *Synechococcus* 11801 has not been performed yet and the findings from this study may open up new avenues to develop cyanobacteria cell factories with augmented metabolic fitness.

## Results

**Figure 2:**
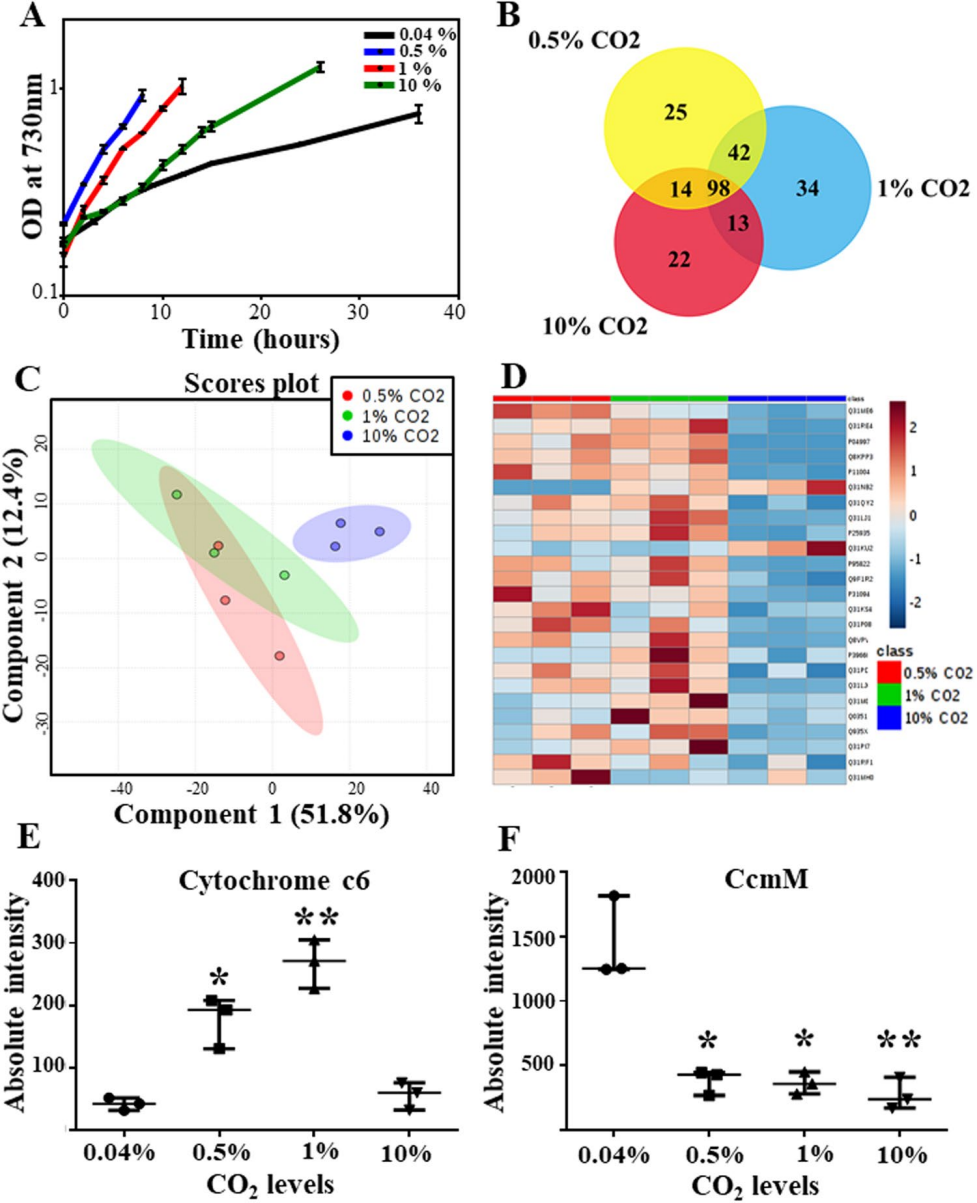
Bioinformatics analysis of differentially expressed proteins. (**A**) Growth curve of *Synechococcus* 11801 at different CO_2_ concentrations at 38 °C and 400 µE in shake flasks. (**B**) Venn diagram showing the distribution of differentially expressed proteins among the three different CO_2_ conditions (0.04%, 0.5% and 10% CO_2_). (**C**) PLS-DA plot is showing segregation among the three high CO_2_ groups based on the protein fold ratio values in each group. The three points in each data group represent the three independent biological replicates. (**D**) Heatmap demonstrating the fold ratio of 25 differentially altered proteins with respect to the expression at ambient CO_2_ levels. (**E**,**F**) Scatter plots with n = 3 (expressed as median with range) showed an overall change in the expression pattern of cytochrome c6 and CcmM protein, respectively, with an increase in CO_2_ levels. Unpaired t-test with Welch’s correction was carried out for the comparison of the three high CO_2_ conditions with the ambient condition, with p-value < 0.05 considered significant. The following p-values were obtained; Cytochrome c6- **0.012** (0.5% CO_2_ vs. 0.04% CO_2_), **0**.**0034** (1% CO_2_ vs. 0.04% CO_2_), **0**.**1993** (10% CO_2_ vs. 0.04% CO_2_) and CcmM- **0**.**0115** (0.5% CO_2_ vs. 0.04% CO_2_), **0**.**0117** (1% CO_2_ vs. 0.04% CO_2_), **0**.**0079** (10% CO_2_ vs. 0.04% CO_2_).

The strain *Synechococcus* 11801 can tolerate up to 15% CO_2_ in the chamber. Its physiological characterization at 0.04% (ambient levels), 0.5%, 1% and 10% CO_2_ resulted in growth rates of 0.07 h^−1^, 0.18 h^−1^, 0.16 h^−1^ and 0.08 h^−1^, respectively^11^ (Fig. 2A). The biochemical characterization under these conditions revealed significant differences in carbon allocation. To understand and validate the molecular and functional mechanisms underlying the process of carbon assimilation, the proteomics study was performed under the respective CO_2_ conditions. It was centered on the rationale that the strain presented a faster growth rate at 0.5% and 1% CO_2_ in comparison to the ambient CO_2_ level of 0.04%. Moreover, after 1% CO_2_, with an escalation in CO_2_ levels, a decline in growth rate was observed. The present study investigates the proteomics changes in *Synechococcus* 11801 under the above-mentioned CO_2_ conditions using the iTRAQ LC-MS/MS assay followed by validation of a few proteins using targeted proteomics-based MRM assays (Fig. 1).

### High CO_2_ induced intracellular modulations elucidated by iTRAQ-based quantitative proteomics study

Three independent biological replicate cultures were cultivated for the iTRAQ experiment and the following labeling strategy was employed uniformly throughout the replicates. The iTRAQ reagents 114, 115, 116 and 117 were used to label protein digests from samples grown at 0.04%, 0.5%, 1% and 10% CO_2_, respectively. The proteomics analysis revealed a total of 861 proteins with at least two peptide signatures, of which 669 proteins were identified in at least two biological replicates. Nonetheless, only those proteins, which were found to be present in all the three replicates, i.e., the 492 proteins (Supplementary Fig. S2) were taken for further investigations. Further, the selection was performed based on the fold ratio values (≥1.5 fold up-regulation or ≤0.66 fold down-regulation) with respect to the ambient condition and for most proteins, the observed differential regulation was seen to be consistent in all three biological replicates. The resulting 248 proteins that showed the same trend (≥1.5 fold up-regulation or ≤0.66 fold down-regulation) across the three replicates were considered for the subsequent statistical and pathway analysis (Supplementary Table S1). These proteins displayed a differential expression pattern in at least one of the three CO_2_ conditions and may serve as vital cues in the understanding of intracellular proteome adjustments in response to elevated CO_2_ levels. It was observed that the protein expression levels at 0.5% and 1% CO_2_ were similar for most of the proteins, as compared to a more drastic protein expression pattern at 10% CO_2_.

### Bioinformatics analysis of differentially expressed proteins during high CO_2_ stress

The protein list provided in Table 1, which includes some of the key differentially expressed proteins was further subjected to PLS-DA (partial least squares-discriminant analysis) using an online tool MetaboAnalyst 4.0^14^ that segregated the three groups (high CO_2_ conditions, viz. 0.5%, 1% and 10% CO_2_) based on the differences among the protein fold ratio values on a 2D-score plot (Fig. 2C). Further, a heatmap was generated displaying the differential expression (fold ratio pattern) of a few of these proteins and is presented in Fig. 2D. Scatter plots of two such proteins showing a significantly altered expression at different CO_2_ levels have been provided in Fig. 2E,F.

**Table 1:** A representative list of proteins from the iTRAQ data (mean ± standard error) showing a differential expression pattern with respect to ambient condition.

Acc. ID	Locus tag	Protein name	0.5%/0.04% CO_2_	1%/0.04% CO_2_	10%/0.04% CO_2_
**Inorganic carbon uptake and assimilation**					
Q03513	DOP62_10195	Carbon dioxide concentrating mechanism protein CcmM	0.09 ± 0.02	0.2 ± 0.05	0.07 ± 0.01
Q03511	DOP62_00505	Carbon dioxide-concentrating mechanism protein CcmK	0.11 ± 0.02	0.1 ± 0.04	0.13 ± 0.1
Q31NB2	DOP62_10175	Ribulose 1,5-bisphosphate carboxylase small subunit	0.03 ± 0	0.07 ± 0.01	0.09 ± 0.01
Q9F1R2	NA	HTH-type transcriptional activator CmpR	0.48 ± 0.06	NS	0.12 ± 0.05
Q55107	DOP62_10270	Bicarbonate transport ATP-binding protein CmpC	0.08 ± 0.04	0.08 ± 0.03	0.04 ± 0.01
P39660	DOP62_10280	Bicarbonate-binding protein CmpA	0.02 ± 0	0.04 ± 0.01	0.02 ± 0
Q8VPV7	DOP62_05970	CO_2_ hydration protein	0.27 ± 0.06	0.32 ± 0.08	0.03 ± 0
Q31ME6	DOP62_10705	NAD(P)H-quinone oxidoreductase subunit H	0.29 ± 0.02	0.15 ± 0.01	0.06 ± 0.01
Q31NJ5	DOP62_07505	NAD(P)H-quinone oxidoreductase subunit I	0.11 ± 0	0.13 ± 0.04	0.05 ± 0.02
Q31P07	DOP62_12075	NAD(P)H-quinone oxidoreductase subunit J	0.18 ± 0.06	0.15 ± 0.05	0.08 ± 0.03
Q31P08	DOP62_12070	NAD(P)H-quinone oxidoreductase subunit K	0.43 ± 0.09	0.28 ± 0.1	0.05 ± 0.01
Q31LQ7	DOP62_07410	NAD(P)H-quinone oxidoreductase subunit M	0.15 ± 0.06	0.26 ± 0.04	0.14 ± 0.02
Q31L05	DOP62_04405	NAD(P)H-quinone oxidoreductase subunit N	0.29 ± 0.09	0.22 ± 0.03	0.07 ± 0.05
**Nitrogen absorption and transport**					
P38043	NA	Nitrate transport protein NrtA	13.9 ± 5.15	11.41 ± 3.7	8.68 ± 3.08
Q31PU9	DOP62_11810	Glutamate synthase (Ferredoxin)	4.56 ± 1.54	3.8 ± 1.42	NS
Q31L83	NA	Glutamine synthetase	11.8 ± 2.14	7.91 ± 1.61	6.17 ± 2
P39661	DOP62_10965	Ferredoxin–nitrite reductase	6.88 ± 2.72	7.11 ± 2.44	7.67 ± 2.63
**Photosynthesis and carbon fixation**					
Q31LJ0	DOP62_06080	Photosystem I P700 chlorophyll a apoprotein A1	3.57 ± 0.72	4.52 ± 1.44	0.38 ± 0.05
Q31LJ1	DOP62_06085	Photosystem I P700 chlorophyll a apoprotein A2	5.2 ± 0.56	8.3 ± 1.92	NS
Q31PI7	DOP62_06380	Photosystem I reaction center subunit II	NS	6.36 ± 2.36	NS
Q31NT9	DOP62_10920	Photosystem I reaction center subunit III	4.47 ± 1.37	5.91 ± 2.28	NS
Q31NL7	DOP62_08820	Photosystem I reaction center subunit IV	4.55 ± 2.48	8.9 ± 4.09	0.12 ± 0.03
P95822	DOP62_05055	Photosystem I reaction center subunit XI	5.9 ± 1.39	7.21 ± 1.56	NS
Q31M07	DOP62_09985	Photosystem II 12 kDa extrinsic protein	NS	3.75 ± 1.28	NS
P11004	DOP62_13040	Photosystem II CP43 reaction center protein	2.71 ± 0.51	2.28 ± 0.2	0.31 ± 0.06
P31094	DOP62_12825	Photosystem II CP47 reaction center protein	NS	1.81 ± 0.18	0.25 ± 0.08
Q31RE4	DOP62_00965	Photosystem II lipoprotein Psb27	2.25 ± 0.19	3.73 ± 0.53	0.33 ± 0.11
P11472	DOP62_00570	Photosystem II manganese-stabilizing polypeptide	NS	NS	0.38 ± 0.09
P04997	DOP62_11795, DOP62_01605	Photosystem II protein D1 2	NS	2.21 ± 0.15	0.07 ± 0.01
Q31RG2	DOP62_01060	Phycobilisome 7.8 kDa linker polypeptide, allophycocyanin-associated, core	14.39 ± 4.17	14.09 ± 4.39	5.42 ± 1.99
Q31PD9	DOP62_11780	Phycobilisome rod linker polypeptide	2.67 ± 0.44	2.93 ± 0.54	NS
P55020	DOP62_06590	Plastocyanin	10.27 ± 3.38	13.63 ± 1.63	9.09 ± 2.69
Q8KPP3	DOP62_12050	Cytochrome b559 subunit alpha	2.9 ± 0.35	3.13 ± 0.61	0.14 ± 0.04
Q54711	DOP62_05005	Cytochrome b6	6.23 ± 2.53	NS	NS
P25935	DOP62_11225	Cytochrome c6	19.64 ± 3.81	30.36 ± 5.91	NS
P0A2Z8	DOP62_04935	ATP synthase epsilon chain	11.66 ± 0.93	13.46 ± 1.27	12.98 ± 6.2
Q31RF0	DOP62_01000	ATP synthase gamma chain	4.61 ± 0.97	2.57 ± 0.22	NS
Q31RF1	DOP62_01005	ATP synthase subunit alpha	11.82 ± 1.87	8.03 ± 1.78	4.35 ± 1.91
Q31RF3	DOP62_01015	ATP synthase subunit b	5.53 ± 2.49	5.54 ± 1.59	3.75 ± 0.31
Q31RF4	DOP62_01020	ATP synthase subunit b’	14.38 ± 3.66	12.09 ± 2.31	8.96 ± 2.19
Q31KS4	DOP62_04930	ATP synthase subunit beta	9.82 ± 1.5	6.4 ± 1.2	3.73 ± 0.76
Q31RF5	DOP62_01025	ATP synthase subunit c	NS	NS	6.5 ± 4
Q31QY2	DOP62_00835	Sedoheptulose 1,7-bisphosphatase	2.51 ± 0.4	2.87 ± 0.37	NS
Q9R6W2	DOP62_10710	Glyceraldehyde-3-phosphate dehydrogenase	5.79 ± 1.17	4.53 ± 0.68	NS
Q31QU9	DOP62_00660	Transketolase	5.31 ± 1.48	3.31 ± 0.85	2.58 ± 0.83
Q935X4	DOP62_03705	Light-dependent NADPH-protochlorophyllide oxidoreductase	7.73 ± 2.53	10.59 ± 2.72	NS
Q31PM2	DOP62_07035	Porphobilinogen deaminase	16.32 ± 5.01	20.42 ± 3.96	16.21 ± 2.13
P16891	DOP62_06580	Uroporphyrinogen decarboxylase	11.55 ± 1.97	8.03 ± 2.75	6.53 ± 1.38
Q31QQ4	DOP62_00020	Mg-protoporphyrin IX chelatase	9.03 ± 1.92	6.69 ± 1.66	5.4 ± 0.35
Q9Z3G6	DOP62_06830	Heme oxygenase (Decyclizing)	3.59 ± 0.88	3.53 ± 0.83	3.08 ± 0.65
Q31LA2	DOP62_05845	Hydrogenobyrinic acid a,c-diamide cobaltochelatase	7.13 ± 1.58	10.26 ± 1.48	5.26 ± 1.89
Q31QJ2	DOP62_13090	Glutamate-1-semialdehyde 2,1-aminomutase	10.02 ± 3.29	9.95 ± 2.3	6.56 ± 2.61
**Photoprotection and ROS response**					
Q31M79	DOP62_09835	Flavoprotein	0.25 ± 0.05	0.16 ± 0.04	0.15 ± 0.03
Q31NJ4	DOP62_07510	NADH dehydrogenase subunit 6	NS	0.34 ± 0.16	0.18 ± 0.08
Q31KG4	DOP62_03465	Chaperon-like protein for quinone binding in photosystem II	NS	0.49 ± 0.02	0.33 ± 0.08
Q7X4K8	DOP62_04890	Thioredoxin peroxidase	0.27 ± 0.02	0.25 ± 0.11	0.24 ± 0.11
Q31MK3	DOP62_11435	Thiosulphate-binding protein	0.27 ± 0.03	0.18 ± 0.07	0.13 ± 0.04
Q79PF2	DOP62_09040	Glutathione peroxidase	0.26 ± 0.1	NS	NS
Q31Q49	DOP62_12620	Glutathione S-transferase	0.17 ± 0.06	0.2 ± 0.08	0.19 ± 0.08
Q31QJ5	DOP62_13105	Bacterioferritin comigratory protein	0.14 ± 0.07	0.35 ± 0.17	0.4 ± 0.14
Q31L59	DOP62_05365	Bacterioferritin comigratory protein	0.35 ± 0.15	0.37 ± 0.01	NS
**Glycolysis**, **TCA cycle and pentose phosphate pathway**					
Q31R18	DOP62_01165	2,3-bisphosphoglycerate-independent phosphoglycerate mutase	3.87 ± 0.67	5.65 ± 1.7	5.66 ± 0.96
Q31NZ1	DOP62_09015	Dihydrolipoyl dehydrogenase	3.45 ± 1.26	2.26 ± 0.33	4.06 ± 1.41
Q31MH0	DOP62_07935	Isocitrate dehydrogenase [NADP]	NS	0.25 ± 0.04	0.34 ± 0.1
P21577	DOP62_01965	6-phosphogluconate dehydrogenase, decarboxylating	0.12 ± 0.03	0.08 ± 0.01	0.16 ± 0.02
Q31KU2	DOP62_04740	Transaldolase	0.25 ± 0.03	0.22 ± 0.03	NS
**Response to high CO**_**2**_ **induced cellular stress**					
P22880	DOP62_04920	10 kDa chaperonin	7.5 ± 3.65	10.31 ± 2.92	10.53 ± 1.3
P22879	DOP62_04915	60 kDa chaperonin	4.93 ± 0.87	4.32 ± 0.75	5.25 ± 0.42

The accession IDs belong to the *Synechococcus* 7942 UniProt FASTA file. Additionally, the locus tags of *Synechococcus* 11801 proteins have been mentioned in the table. *NA = Not found, NS = Not significant.

### Validation of a few key proteins identified from iTRAQ data using MRM

**Figure 3:**
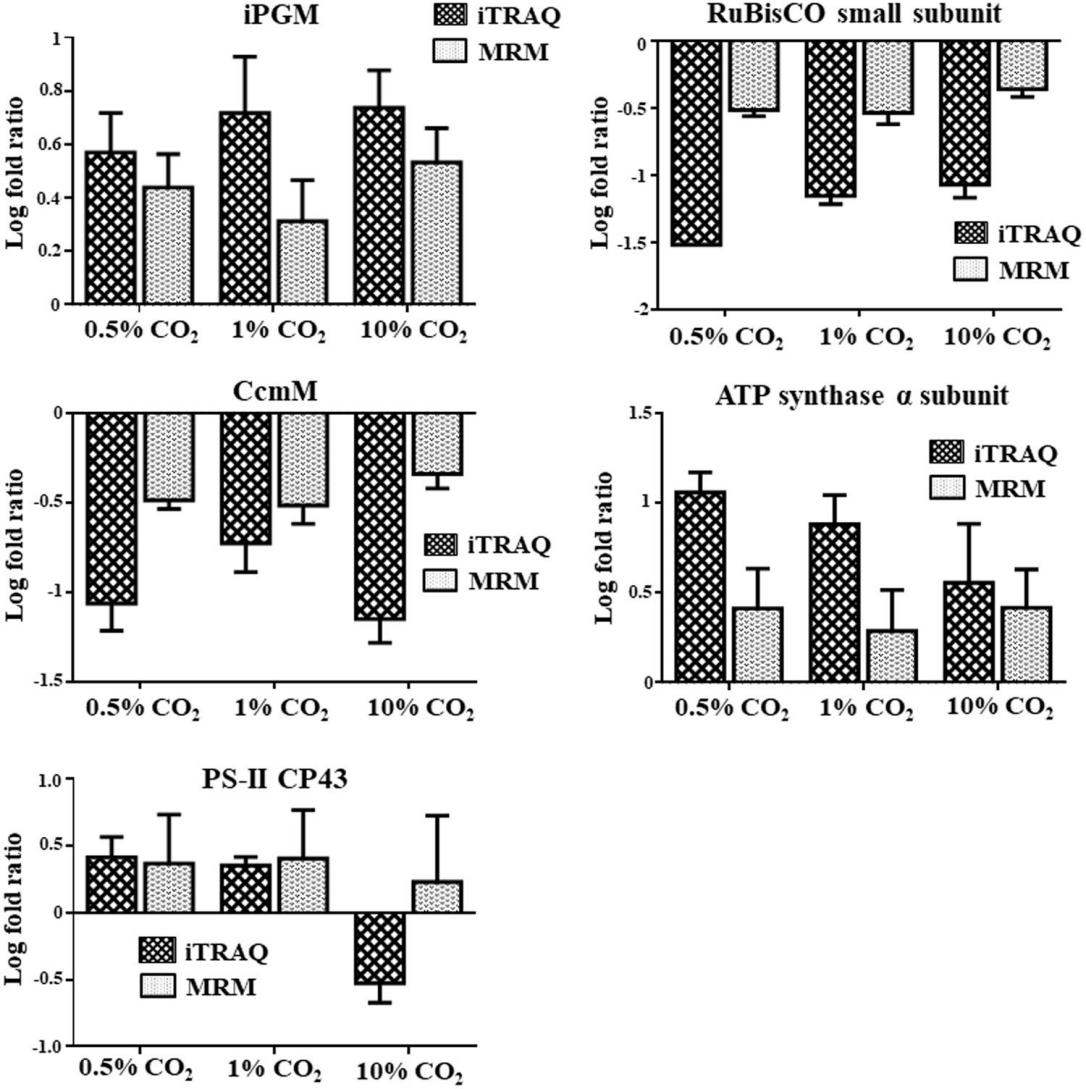
Validation of a few key proteins showing differential expression in iTRAQ data. (**A**) MRM-based relative quantification of iPGM, RuBisCO small subunit, CcmM, ATP synthase α subunit and PS-II CP43 have been shown. The log fold ratio values expressed as mean ± standard deviation (n = 3) were compared with the iTRAQ data and a similar trend was observed for most proteins.

Ten proteins, namely RuBisCO (ribulose-1,5-bisphosphate carboxylase small subunit), carbon dioxide concentrating mechanism protein CcmM, bacterioferritin comigratory protein, SBPase (sedoheptulose 1,7-bisphosphatase), geranylgeranyl reductase, iPGM (2,3-bisphosphoglycerate-independent phosphoglycerate mutase), 60 kDa chaperonin, photosystem II CP43 reaction center protein, ATP synthase α subunit and NrtA (nitrate transport protein) were selected and monitored in all the four CO_2_ condition samples for MRM analysis. The relative expression levels of these proteins were compared with the iTRAQ quantification values. Both the techniques (iTRAQ and MRM) showed a similar trend in expression levels for most of the proteins, which added more confidence to our data. Proteins like ATP synthase α subunit, iPGM, 60 kDa chaperonin showed an up-regulated expression in both iTRAQ and MRM data (Fig. 3 & Supplementary Fig. S3). Similarly, the down-regulated proteins like CcmM, RuBisCO showed a similar expression trend in both the techniques (Fig. 3). However, for a few proteins like PS-II CP43, the two techniques showed a different trend at 10% CO_2_. Previous proteomics studies have also reported discrepancies in the expression levels obtained from iTRAQ and MRM analysis^15,16^. The possible reasons include low abundance of peptides, small sample size and complicated procedure of proteomics analysis without suitable internal standards.

### Pathway mapping of differentially expressed proteins

**Figure 4:**
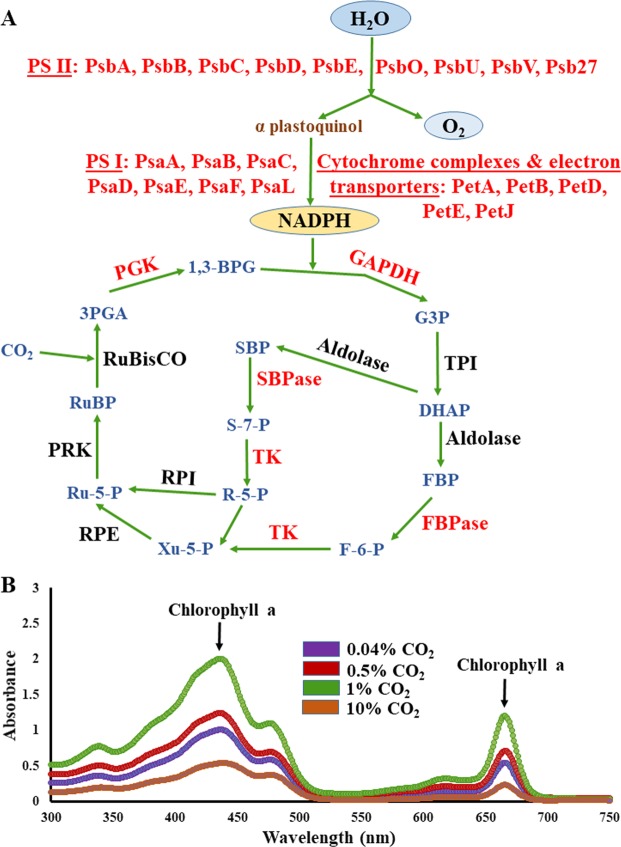
Effect on photosynthetic machinery with an increase in CO_2_. (**A**) Regulation of photosynthetic complexes and Calvin cycle enzymes upon an increase in CO_2_. The proteins highlighted in red showed a differentially altered expression in iTRAQ data. PsbA: Photosystem II reaction center D1 protein, PsbB: Photosystem II CP47 reaction center protein, PsbC: Photosystem II CP43 reaction center protein, PsbD: Photosystem II reaction center D2 protein, PsbE: Photosystem II cytochrome b559 subunit alpha, PsbO: Photosystem II manganese-stabilizing polypeptide, PsbU: Photosystem II 12 kDa extrinsic protein, PsbV: Photosystem II cytochrome c550, Psb27: Photosystem II lipoprotein, PsaA: Photosystem I chlorophyll a apoprotein A1, PsaB: Photosystem I chlorophyll a apoprotein A2, PsaC: Photosystem I iron-sulfur center, PsaD: Photosystem I reaction center subunit II, PsaE: Photosystem I reaction center subunit IV, PsaF: Photosystem I reaction center subunit III, PsaL: Photosystem I reaction center subunit XI, PetA: Cytochrome f, PetB: Cytochrome b6, PetD: Cytochrome b6-f complex subunit 4, PetE: Plastocyanin, PetJ: Cytochrome c6, GAPDH: Glyceraldehyde 3-phosphate dehydrogenase, G3P: Glyceraldehyde 3-phosphate, TPI: Triosephosphate isomerase, DHAP: Dihydroxyacetone phosphate, FBP: Fructose-1,6-bisphosphate, FBPase: Fructose-1,6-bisphosphatase, F-6-P: Fructose-6-phosphate, TK: Transketolase, Xu-5-P: Xylulose 5-phosphate, RPE: Ribulose-phosphate 3-epimerase, Ru-5-P: Ribulose 5-phosphate, PRK: Phosphoribulokinase, RuBP: Ribulose-1,5-bisphosphate, RuBisCO: Ribulose-1,5-bisphosphate carboxylase/oxygenase, 3PGA: 3-phosphoglycerate, PGK: phosphoglycerate kinase, 1,3-BPG: 1,3-Bisphosphoglycerate, SBP: Sedoheptulose 1,7-bisphosphate, SBPase: Sedoheptulose 1,7-bisphosphatase, S-7-P: Sedoheptulose 7-phosphate, R-5-P: Ribose 5-phosphate, RPI: Ribose phosphate isomerase. (**B**) The UV-visible spectra recorded with chlorophyll extracts of the *Synechococcus* 11801 cells grown under 0.04% (ambient), 0.5%, 1% and 10% CO_2_. The peaks corresponding to the absorption maxima of chlorophyll a (435 and 665 nm) are highlighted.

KEGG (Kyoto Encyclopedia of Genes and Genomes) was used to map the 248 differentially expressed proteins to several metabolic pathways and regulatory mechanisms. Major perturbations were observed in cellular transport, central metabolic pathways including glycolysis, PPP (pentose phosphate pathway), TCA cycle (tricarboxylic acid cycle), photosynthesis and carbon fixation (Table 1). Additionally, the expression of several photosynthetic complexes (Fig. 4A) and enzymes belonging to the chlorophyll biosynthesis pathway was perturbed at high CO_2_. To confirm if the chlorophyll amount was increased under high CO_2_ stress, we determined its levels using a UV-visible spectrophotometer. There was a progressive increase in chlorophyll levels with the rise in CO_2_ levels until 1% CO_2_. At 10% CO_2,_ a decline was observed with respect to the ambient condition (Fig. 4B), consistent with the proteomics results which also revealed a decline in photosynthetic machinery at 10% CO_2_.

## Discussion

*Synechococcus* 11801 exhibits a high level of tolerance to different environmental stresses (CO_2_, light, temperature, and salts) and seems like an encouraging prospect for the production of fuels and other industrially relevant chemicals. Its ability to thrive at exceedingly high CO_2_ levels was the foundation of our study, which aims at elucidating the cellular mechanism adopted by cyanobacteria in sequestering high levels of CO_2_. UniProt protein database of the closest neighbour model strain, *Synechococcus* 7942 that shows 90% conservation with *Synechococcus* 11801 at the proteome level^11^, was used for the proteomics analysis. The global proteomics analysis revealed modulations among diverse protein groups and for a better understanding of their roles, they have been categorized into small sub-groups, which are discussed in detail hereafter.

### Inorganic carbon uptake and assimilation

One of the most drastic responses towards high CO_2_ levels comprised of a diminished synthesis of regulatable CCM components involved in the uptake of Ci (inorganic carbon) and enhanced production of nitrogen assimilatory proteins as a feedback mechanism. This decrease in CCM components in cyanobacteria and microalgae at high CO_2_ is a well-established phenomenon^17,18^. CCM has evolved to expand the photosynthetic performance of cyanobacteria and factors like CO_2_ availability strongly influence the biosynthesis of many of its components^19^ including the five distinct transporters for active Ci uptake^20^; (i) BCT1, an ABC (ATP-binding cassette) bicarbonate transporter encoded by the *cmpABCD* operon^21^, (ii) SbtA, a high-affinity sodium-dependent bicarbonate transporter^22^ (iii) BicA, a low-affinity sodium-dependent bicarbonate transporter^23^ (iv) NDH-I_4_, a low-affinity CO_2_ uptake system constitutively expressed and (v) NDH-I_3_, a high-affinity CO_2_ uptake system inducible under limiting CO_2_ conditions^24,25^. CCM provides a growth advantage, predominantly under CO_2_-limiting conditions wherein the expression levels of inorganic carbon transporters have been reported to be augmented in several omics reports^26–29^. On the contrary, another proteomics study that was performed to understand the effect of high CO_2_ on cyanobacteria has revealed that the expression of CCM related proteins is suppressed at high CO_2_ levels^12^. This is in accordance with our study wherein we have found a substantial down-regulation of CmpA and CmpC protein at all three high CO_2_ concentrations, i.e., 0.5%, 1% and 10% (Table 1). The *cmpABCD* operon is activated under CO_2_-limited conditions by CmpR, a LysR family transcription regulator^30^, which too showed a down-regulation in our study. ChpY, a CO_2_ hydration protein which is a part of the high-affinity CO_2_ uptake NDH-I_3_ complex^25^ also showed a uniform decreased expression with respect to the ambient condition.

Correspondingly, CcmK and CcmM the structural components of carboxysomes which are essential for its assembly and functioning^31,32^ were seen to be down-regulated in all three CO_2_ concentrations. Also, the expression of carboxysomal proteins like carbonic anhydrase and RuBisCO small subunit was considerably diminished with the increase in CO_2_ concentration. Since carboxysomes house most of the cellular RuBisCO, the decline in shell components (CcmK, CcmM) is in agreement with the down-regulation of the enzyme. Interestingly, carbonic anhydrase in plants is also known to exhibit antioxidant activity and plays an additional role in the defense response^33^. Expression of several subunits of the NDH-1 complex (which is known to be involved in cellular functions like respiration, carbon dioxide uptake, etc.) was also found to be down-regulated at all three high CO_2_ levels. A recent study by Hirokawa and co-workers had shown improved productivity of 1,3-propanediol (1,3-PDO) in *Synechococcus* 7942 by disrupting the gene encoding NDH-1 complex subunits^34^. By eliminating the complex, they observed an increased carbon flux into the Calvin cycle demonstrating it as an attractive route towards an enhanced production of 1,3-PDO and glycerol, further suggesting a suitable target for metabolic engineering.

### Nitrogen transport and absorption

It is imperative for cyanobacteria to maintain a harmony between carbon and nitrogen levels for the optimal cell metabolism, therefore, amplified levels of nitrogen assimilatory proteins could be perceived as an attempt to balance the carbon-nitrogen ratio (Table 1). At high CO_2_, elevated expression of NrtA, NrtC (nitrate transport ATP-binding protein), glutamate synthase (ferredoxin), glutamine synthetase III, glutamine synthetase and ferredoxin–nitrite reductase was observed in a possible effort to maintain the homeostasis of C:N ratio.

The major nitrogen sources for cyanobacteria include ammonium, nitrate, nitrite and urea of which nitrate is predominantly present in the BG-11 medium^35^. Nitrate assimilation in cyanobacteria encompasses three sequential steps; (i) nitrate uptake into the cell through an ABC-type transporter (that constitutes the products of the *nrtA*, *nrtB*, *nrtC* and *nrtD* genes)^36–38^, (ii) a two-step conversion to ammonium in a sequential manner by enzymes NarB (ferredoxin-nitrate reductase) and NirA (ferredoxin nitrite reductase)^39,40^ and (iii) the incorporation of resulting ammonium generated from nitrate reduction into carbon skeletons via the GS-GOGAT (glutamine synthetase-glutamate synthase) pathway^38,41^. Moreover, the transporter-encoding genes in *Synechococcus* 7942 are clustered together and co-transcribed with *nirA* and *narB* genes constituting the operon *nirA-nrtABCD-narB*^42,43^. Previous studies have delineated stimulatory effects of inorganic carbon on the synthesis of nitrate reductase emphasizing how nitrate utilization is strongly predisposed to the availability of CO_2_^44^. Moreover, a CO_2_ enriched environment in *Hizikia fusiforme* culture boosted nitrate uptake and the activity of nitrate reductase^45^, further corroborating our findings. In our study, though the expression of NrtA remained exceedingly high in all the conditions, a trend was observed with the increase in CO_2_ levels as it showed an up-regulation of 13.9 folds in 0.5%, 11.4 folds in 1% and 8.7 folds in 10% CO_2_ condition. On the other hand, NrtC exhibited a significant increase of 3.7 folds only in 10% CO_2_ condition. The enzymes involved in GS-GOGAT pathway which is reported to be favored in ammonium-limiting conditions or under high energy supply^46,47^, viz. glutamine synthetase and ferredoxin-dependent glutamate synthase, were also found to be elevated in our study. Despite that the contents of growth medium were the same for ambient and high CO_2_ samples, it is understandable that high CO_2_ environment favors biosynthesis of nitrogen assimilatory proteins as a part of the acclimatization mechanism.

In addition to carbon and nitrogen, expression levels of few other ABC transporters involved in transmembrane ion transport were found to be regulated, i.e., an up-regulation of phosphate binding protein along with a decline in the expression of iron and sulfate-binding protein was observed.

### Photosynthesis and carbon fixation

The growth of *Synechococcus* 11801 at high CO_2_ levels resulted in a steep rise in its cellular photosynthetic activity wherein an overall surge in expression was observed for photosynthetic proteins, which could be due to the abundant inorganic substrate available for carbon-fixing reactions. Key proteins identified in our study include cytochromes, plastocyanin, reaction centers of PS-I and PS-II (photosystem-I and II), etc. but interestingly few of these proteins showed a significant downshift at 10% CO_2_. Light-harvesting phycobilisome components like the alpha subunit of allophycocyanin was 3.6 folds up-regulated in 1% CO_2_ condition whereas the beta subunit showed a 0.17 folds down-regulation at 10% CO_2_. Previously a proteomics study on *Synechocystis* 6803 had reported slight down-regulation of subunits of PS-I, PS-II and phycobilisomes upon CO_2_ downshift^26^. Similarly, the transcriptomics response of *Synechocystis* 6803 to carbon limitation studied by Wang and co-workers too revealed a reduced expression of PS-I and PS-II genes^28^. They further suggested that this reduced requirement for the products of light reactions is due to the limited inorganic substrate available for carbon-fixing reactions.

Interestingly, carbon dioxide has been hypothesized to directly regulate the photosynthetic apparatus in a number of ways, namely by bicarbonate’s involvement in electron donation for the donor side of PS-II^48^ or by bicarbonate’s role as a proton shuttle stabilizing the binding niche of quinone, stimulating plastoquinol formation^49^ or by the activation of RuBisCO that requires CO_2_ (distinct from the CO_2_ that is fixed) before catalysis^50–52^. Previous studies also suggest that a short-term CO_2_ enrichment stimulates photosynthetic rate with a concomitant increase in biomass whereas prolonged exposure to high CO_2_ may suppress photosynthesis^53^. Though in our study we subjected the cells to high CO_2_ stress instead of prolonged exposure to CO_2_, few photosynthetic proteins showed a strong down-regulation at 10% CO_2_. Rather than being a direct response to high CO_2_, this suppression may be attributed to secondary responses linked to carbohydrate accumulation. With the photosynthetic rate exceeding the capacity to utilize the photosynthate for cellular growth, carbohydrate accrual may result into the repression of photosynthetic genes due to a probable feedback inhibition mechanism^53^. Additionally, reduced nitrogen and RuBisCO content may also contribute to the repression of photosynthesis.

Correspondingly, the proteins associated with carbon fixation like SBPase, glyceraldehyde-3-phosphate dehydrogenase and transketolase were up-regulated at elevated CO_2_ to facilitate fixation of the copious amount of inorganic carbon available. On numerous occasions, it has been suggested that under an array of growth conditions SBPase exerts a profound control over the Calvin cycle^54^ and also by increasing the SBPase activity, the photosynthetic rates were markedly increased in plants like tomato and tobacco^55,56^ suggesting that a higher SBPase activity may be advantageous for photosynthesis and growth. Even slight reductions in SBPase activity results in a substantial decrease in photosynthetic rates^57,58^, rendering further evidence that SBPase is tightly linked to carbon flux in the Calvin cycle. The enzyme functions at a branch point where assimilated carbon may either be used to regenerate the CO_2_ acceptor molecule ribulose-1,5-bisphosphate in the Calvin cycle or be assimilated for starch or sucrose and this precise position signifies the prominence of SBPase in the regulation of carbon flow. On the contrary, initial attempts focused at ameliorating carbon fixation were centered on modifying the catalytic properties of RuBisCO, as it was believed to be the key rate-limiting step in the overall photosynthetic incorporation of carbon^59,60^. However, later it was observed that the levels of RuBisCO had less influence on the control of carbon fixation under a wide range of environmental settings, especially under saturating CO_2_ conditions, so much so that even halving the activity of RuBisCO did not lead to any substantial diminution in the photosynthetic capacity^54^. Another protein found in our study, glyceraldehyde-3-phosphate dehydrogenase (encoded by *gap2* gene), is essential for the Calvin cycle and is distinct from the other glycolytic enzyme glyceraldehyde-3-phosphate dehydrogenase involved in catabolic glucose degradation^61,62^. Likewise, transketolase’s expression levels were seen to be augmented in our findings because the enzyme apart from taking part in the pentose phosphate pathway is also involved in carbon fixation. An interesting study by Liang *et al*. has reported an increase in the growth rate and biomass of *Synechocystis* 6803 upon over-expression of proteins like SBPase and transketolase, highlighting them as potential candidates for an improved growth^63^.

Akin to proteins of photosynthetic machinery, enzymes involved in the biosynthesis of cofactors such as chlorophyll and porphyrin were markedly up-regulated at high CO_2_, namely light-dependent NADPH-protochlorophyllide oxidoreductase, uroporphyrinogen decarboxylase, Mg-protoporphyrin IX chelatase, porphobilinogen deaminase, heme oxygenase and hydrogenobyrinic acid a,c-diamide cobaltochelatase (Table 1). These results were found to be consistent with the chlorophyll levels estimated under different CO_2_ conditions (Fig. 4B). This increase in the synthesis of pigments and cofactors that play a pivotal role in the uptake of light mirrors the undergoing acclimation activity associated with magnified photosynthetic rates. The protein geranylgeranyl reductase, which is involved in chlorophyll and tocopherol biosynthesis^64,65^ demonstrated a marked 2.33 folds up-regulation at 0.5% CO_2_ and 0.21 folds down-regulation at 10% CO_2_. Apart from photosynthesis, proteins belonging to oxidative phosphorylation like ATP synthase also showed pronounced up-regulation at all three high CO_2_ conditions. All the different subunits of ATP synthase that belong to the same functional complex showed an enhanced expression adding high confidence to the acquired data.

### Photoprotection and ROS (reactive oxygen species) response

Flavoprotein (Ortholog of Flv1 found in *Synechocystis* 6803) that plays a critical role in maintaining cellular redox homeostasis was found to be significantly down-regulated in all three high CO_2_ conditions (Table 1). It is known to catalyze photoreduction of O_2_ to H_2_O without producing ROS at the acceptor side of PS-I, ultimately protecting the oxygen-evolving PS-I complex against photooxidative damage^66^. Other antioxidant enzymes that protect cells against ROS, for example, glutathione peroxidase, thioredoxin peroxidase, superoxide dismutase, glutathione S-transferase, also demonstrated a decline in their expression. An important group of cyanobacteria proteins that catalyze the reduction of hydroperoxides and confer resistance to oxidative stress is peroxiredoxin^67^. 1-Cys peroxiredoxin, which is hypothesized to be involved in protecting nucleic acids against oxidative damage^68^ showed a 0.33 and 0.23 folds down-regulation at 0.5% and 10% CO_2_, respectively. Bacterioferritin comigratory protein, classified as atypical 2-Cys peroxiredoxin^67^, which in a previous study along with glutathione peroxidase-reductase system has been shown to be responsible for the detoxification of bentazone-derived peroxides also displayed a strong downshift in our findings^69^. Another peroxidase found in our study, viz. thioredoxin peroxidase was identified by Yamamoto and co-workers in *Synechocystis* 6803 as an antioxidant protein involved in reducing H_2_O_2_ with an electron transport system-coupled cellular activity^70^. An excess of excitation energy damages components of the photosynthetic machinery and produces harmful ROS, particularly when anabolism is slowed down due to nutrient deprivation^68^ and thus photoprotection mechanism is generally enhanced to protect cells from any possible photodamage. This resonates with a comparative proteomics study carried out by Gao *et al*. wherein they observed an extreme ROS formation by photosynthetic pigments and redox components when *Synechocystis* 6803 cells were treated with prolonged UV-B (Ultraviolet-B) radiation leading to a reduced light-harvesting ability and an increased expression of antioxidant proteins^71^. Also, there are numerous studies on plants in which the shared defense mechanisms against environmental pressures like metal toxicity, drought, salinity, chilling, UV-B radiations, etc. lead to increased activity/levels of these antioxidant proteins in response to the enhanced ROS production^72,73^. On the contrary, in our study, since at high CO_2_ levels, most of the captured light is utilized in carbon fixation, maintaining a balance between light capture and its utilization, an underproduction of ROS stress-responsive enzymes was observed.

### Glycolysis, TCA cycle and PPP

The *Synechococcus* 11801 cells at high CO_2_ exhibited a heightened glycolysis pathway with a downshift in the TCA cycle and PPP. Expression levels of glycolytic enzymes belonging to the pay-off phase including phosphoglycerate kinase, 2,3-bisphosphoglycerate-independent phosphoglycerate mutase and pyruvate kinase evinced a strong up-regulation. Another group of proteins that constitute the pyruvate dehydrogenase complex showed an up-regulation at high CO_2_, but enzymes belonging to the TCA cycle, namely isocitrate dehydrogenase and aconitate hydratase displayed a decline in their expression. Since the flux to acetate does not produce any NADH in comparison to the flux from acetyl-CoA through the TCA cycle which generates NAD(P)H and FADH_2_, this diversion of carbon flow to acetate, especially in heterotrophs like *E*. *coli*. is often regarded as an apt measure to avert additional NAD(P)H accretion^74,75^. Analogous to TCA cycle, proteins like 6-phosphogluconate dehydrogenase (decarboxylating), transaldolase, glucose-6-phosphate 1-dehydrogenase showed a marked decline in their expression. The oxidative pentose phosphate pathway is predominantly involved in the production of reducing equivalents like NADPH that maintain cellular redox, especially under any oxidative stress. Since the photoprotection mechanism is already repressed, the cellular state of *Synechococcus* 11801 did not favour the pentose phosphate pathway.

### Other proteins and pathways

Several proteins like farnesyl-diphosphate synthase, geranylgeranyl reductase, acetolactate synthase, ketol-acid reductoisomerase that participate in the biosynthesis of terpenoids and branched-chain amino acids showed an up-regulated expression. Various ribosomal proteins, elongation factors and uncharacterized proteins also showed an altered protein expression at high CO_2_ conditions. Additionally, an up-regulated 3-oxoacyl-[acyl-carrier-protein] synthase 2 protein was seen in our study. This protein catalyzes the condensation reaction of fatty acid synthesis indicating a possible enhanced lipid production, especially since acetyl-coenzyme A synthetase’s expression levels were also found to be increased in our study. High CO_2_ concentrations in microalgae are known to increase the levels of acetyl CoA, which is a precursor of fatty acid biosynthesis, eventually resulting in lipid accumulation^76^. Sun and co-workers had shown that genes involved in triacylglycerol biosynthesis were considerably up-regulated at elevated CO_2_ in *Chlorella sorokiniana*^77^. They further suggested that in microalgae an alternative strategy of providing excess CO_2_ for lipid accumulation may be a preferable approach over the more traditional strategy of nitrogen starvation since nitrogen deprivation may limit cellular growth. In contrast, by growing microalgae under high CO_2_ condition, both higher growth and lipid content can be achieved and since microalgae can assimilate CO_2_ from the industrial flue gases, it may be a more advantageous approach for lipid production at a wider scale. They also discovered that under high doses of CO_2_, expression of genes participating in central carbohydrate metabolic pathways including carbon fixation, glycolysis, and components of the pyruvate dehydrogenase complex were up-regulated, which was consistent with our findings.

At high CO_2_ levels, the *Synechococcus* 11801 cells presented paramount changes in proteins participating in carbon-nitrogen uptake and assimilation, photosynthetic complexes, CO_2_ fixation, glycolysis, TCA cycle, oxidative stress response and translation machinery, as a sequestration prototype towards high CO_2_. Additionally, in our study, cellular stress-responsive proteins like 10 and 60 kDa chaperonins were seen to be up-regulated in high CO_2_ conditions. A large number of uncharacterized proteins were also discovered in the study whose expression levels were notably altered under high CO_2_ conditions. The exact roles played by these unknown proteins remain largely unidentified indicating that our understanding of cyanobacteria’s cellular response towards high CO_2_ levels is still limited. This opens up new dimensions for exploration to fully comprehend the internal homeostatic response to CO_2_ stress in cyanobacteria. Moreover, there is a growing body of evidence showing that rising CO_2_ concentrations can induce rapid adaptive alterations in the genotype composition of photosynthetic species and may even lead to evolutionary changes^78^. It is very well demonstrated that many cyanobacteria including the model strain *Synechococcus* 7942 and its fast-growing neighbour strains like *Synechococcus* 2973 and *Synechococcus* 11801 produce more biomass in the presence of CO_2_^10,11,79^. In a host cyanobacteria, the ability to grow at high CO_2_ conditions is desired as a heterologous expression of any pathway leads to significant redirecting of carbon towards the desired product, generating more demand of carbon that can be fulfilled by cultivating engineered strains in the presence of high CO_2_^4,80,81^. Therefore, the understanding of intracellular alterations using proteomics may shed light on the strategy adopted by cyanobacteria in sequestering excess CO_2_. Our results offer novel insights into the global cellular response towards elevated CO_2_ in the indigenous strain *Synechococcus* 11801, which may be directly extrapolated to future studies addressing CO_2_ sequestration mechanisms in close neighbour strains like *Synechococcus* 7942, *Synechococcus* 2973 and other closely related photosynthetic organisms whose central metabolism is nearly conserved. In conclusion, the present study provides a global view of the differential cellular responses that occur in *Synechococcus* 11801 at the proteome level while going from carbon-limiting to carbon-sufficient conditions. Many proteins from our findings may be taken up as prospective targets for host and pathway engineering in *Synechococcus* 11801 and its close relatives.

## Methods

### Cultivation conditions of *Synechococcus* 11801

The strain *Synechococcus* 11801 was cultured in non-buffered BG-11 medium (initial pH = 7.5) and shake flasks. The culture was grown at 38 °C, 120 rpm and light intensity of 400 µmole photon.m^−2^.s^−1^ in a CO_2_ incubator shaker (Adolf Kuhner AG, LT-X, Birsfelden, Switzerland). The CO_2_ concentrations in the chamber were varied according to the experimental requirements. Three biological replicates of the cells (OD_730_ = 0.8) grown under 0.04%, 0.5%, 1% and 10% CO_2_ were harvested by centrifugation at 8,000 × g for 10 minutes at 4 °C. The cells were washed thrice with ice-cold phosphate buffer, pH = 7.5 and the pellets were stored at −80 °C till use.

### Protein extraction, quantification and quality check

The protocol reported by Guerreiro *et al*.^82^ was used for protein extraction from the bacterial pellet with minor modifications. In brief, the pellet was resuspended in a lysis buffer having 50 mM ammonium bicarbonate, 8 M urea and a protease inhibitor cocktail. The cell lysis was performed by subjecting the samples to five freeze-thaw cycles using liquid nitrogen followed by five cycles of sonication on ice (30% amplitude for 1 min, 2-sec pulse and 2-sec gap). The supernatant was collected, after the centrifugation was carried out at 12,000 × g for 30 minutes and was further used for proteomics experiments. The protein concentration in each sample was estimated using QuickStart Bradford reagent (Bio-Rad, USA) and for quality check, the samples were run on a 12% SDS-PAGE gel. Further, the protein sample was used for iTRAQ-based quantitative proteomics analysis.

### Sample processing for LC-MS/MS analysis (In-solution digestion, iTRAQ labeling and peptide OFFGEL fractionation)

The protein samples extracted from the three biological replicates were used for iTRAQ-based quantitative proteomics analysis. Prior to iTRAQ labeling, 50 µg of the protein sample was taken from all the four conditions, i.e., 0.04% CO_2_, 0.5% CO_2_, 1% CO_2_ and 10% CO_2_ and in-solution digestion was performed using the enzyme trypsin (Trypsin Gold, Mass spectrometry grade, Promega, Madison, WI, USA). The protein samples were diluted with dissolution buffer provided with iTRAQ kit to decrease the urea concentration followed by reduction with TCEP [tris (2-carboxyethyl) phosphine] at 60 °C for 1 hour. Subsequently, the samples were alkylated using MMTS (methyl methanethiosulfonate) for 30 minutes at room temperature to prevent the reformation of disulphide bonds. Trypsin was added at a 1:30 trypsin: protein ratio and the samples were kept at 37 °C for 16 hours for complete protein digestion.

After in-solution digestion, labeling of the digested peptides was performed using a 4plex-iTRAQ reagent multi-plex kit (AB Sciex, USA) as per the manufacturer’s instructions in a manner that iTRAQ reagents 114, 115, 116 and 117 were used to label trypsin-digested peptides from the samples grown at 0.04%, 0.5%, 1% and 10% CO_2_, respectively. The labeling reaction was quenched using 100 μL of water followed by incubation for 30 minutes at room temperature. All the labeled samples of the respective replicate set were pooled together and fractionated using Agilent 3100 OFFGEL fractionator (Agilent Technologies, USA) with IPG strip (pH range: 4–7, length: 24 cm) following manufacturer’s instructions. Each fraction was collected separately, and the peptide mixture was further cleaned up using zip-tips having C-18 columns (Millipore, USA) for salt removal before the MS/MS analysis. The labeled, fractionated, zip-tipped fractions were analyzed using Triple-TOF 5600 instrument and the raw.wiff files obtained were investigated using the ProteinPilot software (5.0.1, AB Sciex).

### LC-MS/MS analysis for the database-aided protein identification and quantitation

The desalted samples were reconstituted in 0.1% formic acid and analyzed on a Triple-TOF instrument (SCIEX, Framingham, MA) coupled to eksigent ekspert^TM^ nanoLC 425. The data acquisition was performed in a positive ion mode wherein the MS and MS/MS spectra were acquired in the range of 350–1250 and 100–1800 m/z, respectively. The instrument was operated in DDA (data-dependent acquisition) mode wherein maximum 20 precursors per cycle were chosen for fragmentation from each MS spectrum. The tandem MS/MS spectra were obtained in a high sensitivity mode with iTRAQ reagent collision energy adjustment. The raw files obtained in the.wiff format were analyzed using the ProteinPilot software and searched against the *Synechococcus* 7942 UniProt database. Paragon^TM^ search algorithm was used for protein identification and quantification^83^. The following parameters were selected for the analysis: Sample type- *iTRAQ 4plex (peptide labeled)*; Cys alkylation- *MMTS*; Digestion- *Trypsin*; Instrument- *Triple TOF 5600*; Special factors- *Urea denaturation*; ID focus- *Biological modifications*. Further, the database search was carried out with a *Thorough effort* while the detected protein threshold confidence was set at 99%, which corresponded to an unused score of 2. The option for FDR (false discovery rate) was also selected (Supplementary Fig. S1). The acquired mass spectrometric data files from the shotgun proteomics experiments have been deposited to the ProteomeXchange Consortium via the PRIDE^84^ partner repository with the dataset identifier PXD011485.

### MRM to validate key findings from the iTRAQ shotgun MS/MS data

Ten differentially expressed proteins from the iTRAQ data, namely RuBisCO, CcmM, bacterioferritin comigratory protein, SBPase, geranylgeranyl reductase, iPGM, 60 kDa chaperonin, PS-II CP43 reaction center protein, ATP synthase α subunit and NrtA were chosen for MRM-based validation. The FASTA file of *Synechococcus* 7942 was fed into the Skyline software^85^ to generate *in silico* trypsin-digested peptides, from which unique peptides to these proteins were filtered. Further refinement was done by choosing precursor and product ions having only +2 and +1 charge, respectively. The peptide length ranged from 8–16 amino acids with no missed cleavages and at least 3 peptides were analyzed from each protein with a minimum of 3 transitions monitored per peptide. A total of 217 transitions were monitored for these 10 proteins in a single method run. Information like peptide sequences, precursor and product m/z, retention time have been provided in Supplementary Table S2. The MRM analysis was carried out on TSQ Altis™ triple quadrupole mass spectrometer (Thermo, USA) coupled to EASY-nLC™ 1200 in a scheduled manner with a retention time window of ± 1.5 minutes. Both the quadrupoles were operated at 0.7 resolution units (FWHM) and a cycle time of 1 second was kept. All the three biological replicates were analyzed and the result files were imported into the Skyline software followed by peak area computation.

### Bioinformatics functional analysis to identify perturbed pathways

Pathway identification of the differentially altered proteins was done using KEGG wherein the UniProt Accession IDs were uploaded and matched against the reference species (*Synechococcus* 7942). Further, the online tool MetaboAnalyst was employed in generating PLS-DA plot and heatmap, which showed the segregation among different CO_2_ conditions based on the protein fold ratio values. Scatter plots were generated using the GraphPad Prism software.

### Measurement of chlorophyll using spectrophotometer

The chlorophyll was extracted from 2 mL of cells at OD_730 nm_ = 0.8 under all the conditions (0.04%, 0.5%, 1% and 10% CO_2_) using a previously described protocol^86^. The spectra were recorded from 300–750 nm using UV-visible spectrophotometer (Shimadzu, UV-2600, Singapore).

## Supplementary information

Supplementary Information
