# Supplementary material for: Elevated carbon dioxide levels lead to proteome-wide alterations for optimal growth of a fast-growing cyanobacterium, Synechococcus elongatus PCC 11801
Source: Sci Rep. 2019 Apr 18;9:6257. doi: 10.1038/s41598-019-42576-1 (PMC6472392; doi:10.1038/s41598-019-42576-1)
Supplement: Supplementary file 1 — Supplementary Information [file 41598_2019_42576_MOESM1_ESM.pdf]

Elevated carbon dioxide levels lead to proteome-wide alterations for optimal growth of a fast growing cyanobacterium, *Synechococcus elongatus* PCC 11801

Kanika Mehta,[1] Damini Jaiswal,[2] Monalisha Nayak,[2] Charulata B. Prasannan,[2,3] Pramod P. Wangikar[2,3,4] and Sanjeeva Srivastava[*1,3,4]

[1]Department of Biosciences and Bioengineering, Indian Institute of Technology Bombay, Powai, Mumbai-400076, India. [2]Department of Chemical Engineering, Indian Institute of Technology Bombay, Powai, Mumbai-400076, India. [3]DBT-Pan IIT Center for Bioenergy, Indian Institute of Technology Bombay, Powai, Mumbai-400076, India. [4]Wadhwani Research Center for Bioengineering, Indian Institute of Technology Bombay, Powai, Mumbai-400076, India. Correspondence and requests for materials should be addressed to S.S. (email: sanjeeva@iitb.ac.in)

Supplementary figures

Fig. S1: Parameters used in ProteinPilot software for protein identification and quantification.

Fig. S2: Venn diagram showing common proteins among the three independent biological sets identified through iTRAQ.

Fig. S3: Validation of few proteins using MRM assay. In most cases same trend was observed for both iTRAQ and MRM but for few proteins at 10% CO_2 level different trends were seen.

Fig. S4: Heatmap illustrating the expression pattern of all the 248 differentially expressed proteins.

Fig. S5: Scatter plots (generated using online BioCyc tool) showing an overall protein expression pattern across different metabolic processes with increasing CO_2 levels.

Fig. S1: Parameters used in ProteinPilot software for protein identification and quantification.

**Search Method**	
Sample Type	iTRAQ 4plex (Peptide Labeled)
Cysteine Alkylation	MMTS
Digestion	Trypsin
Special Factors	Urea denaturation
Instrument	TripleTOF 5600
Species	
Search Effort	Thorough
ID Focus	Biological modifications
FDR Analysis	Yes
Quantitation	
Background Correction	Yes
Bias Correction	Yes
Channel to use as denominator in ratios	
Modified Data Dictionary or Parameter Translation	No
**Database Searched**	
Database filename	C:\AB SCIEX\ProteinPilot Data\SearchDatabases\uniprot-synechococcus+elongatus+pcc+7942.fasta
Number of Proteins in Database	2875
Number of proteins searched	5750

Fig. S2: Venn diagram showing common proteins among the three independent biological sets identified through iTRAQ.

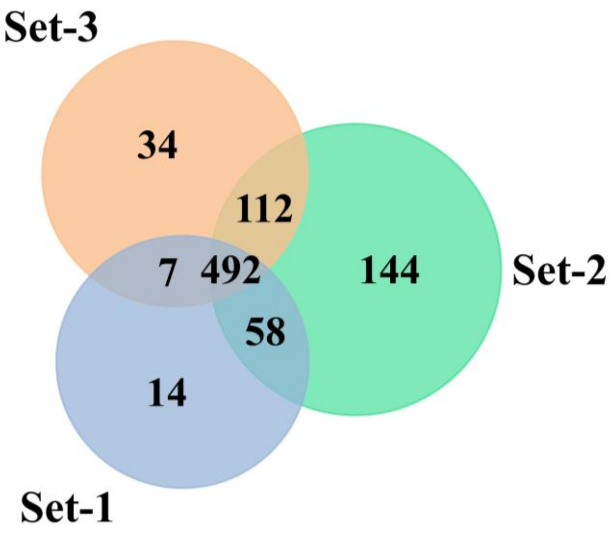

Fig. S3: Validation of few proteins using MRM assay. In most cases same trend was observed for both iTRAQ and MRM but for few proteins at 10% CO_2 level different trends were seen.

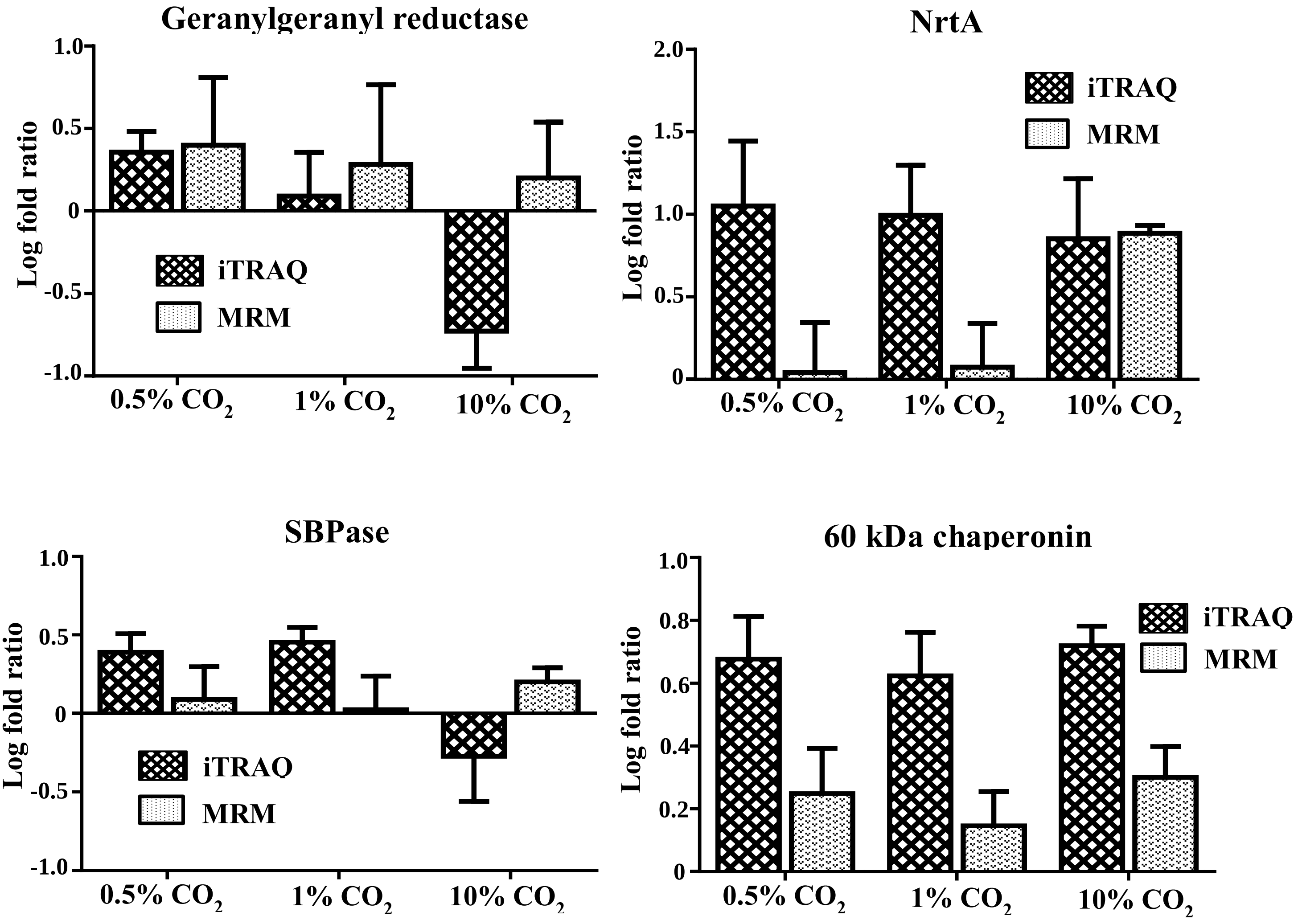

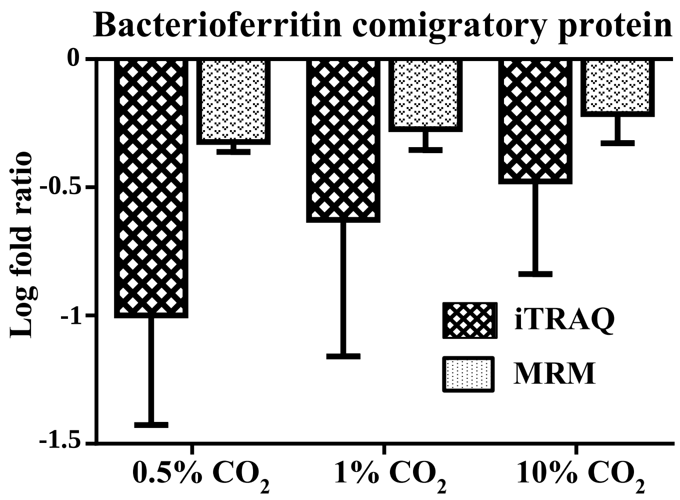

Fig. S4: Heatmap illustrating the expression pattern of all the 248 differentially expressed proteins.

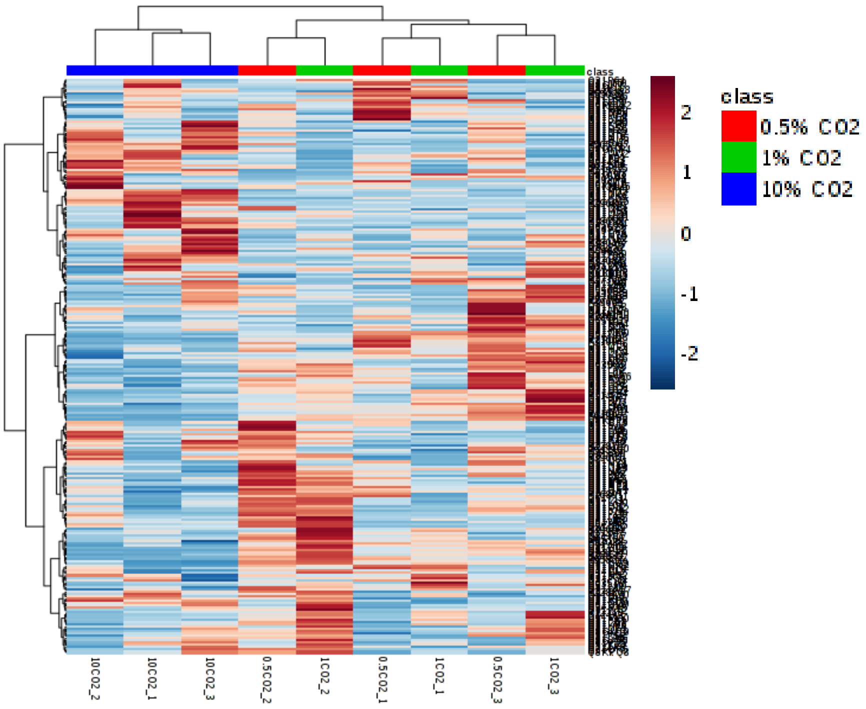

Fig. S5: Scatter plots (generated using online BioCyc tool) showing an overall protein expression pattern across different metabolic processes with increasing CO_2 levels.

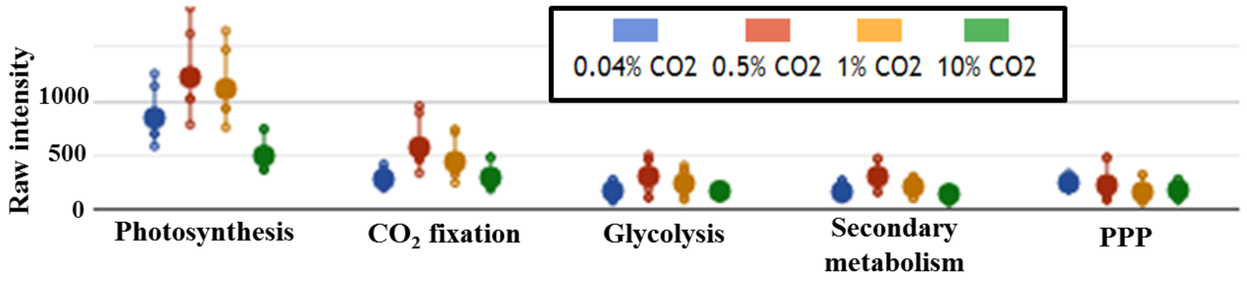

Supplementary tables

Table S1: Fold ratio values (with respect to the ambient condition) of 248 differentially expressed proteins across the three biological sets, depicting the cellular response towards high CO_2 stress.

Table S2: Transition list obtained from Skyline for the MRM assay on TSQ Altis™ triple quadrupole mass spectrometer.

Table S1: Fold ratio values (with respect to the ambient condition) of 248 differentially expressed proteins across the three biological sets, depicting the cellular response towards high CO_2 stress.

Accession	Name	**0.5% CO_2_1/2/3 (Pval_1/2/3)**	**1% CO_2_1/2/3 (Pval_1/2/3)**	**10% CO_2_1/2/3 (Pval_1/2/3)**
Q03513	Carbon dioxide concentrating mechanism protein CcmM	0.06/0.12/0.09 (0/0/0)	0.29/0.15/0.15 (0/0/0.01)	0.08/0.05/0.09 (0/0/0)
Q03511	Carbon dioxide-concentrating mechanism protein CcmK	0.15/0.08/0.11 (0/0/0.08)	0.04/0.18/0.09 (0/0/0.04)	0.02/0.32/0.05 (0.01/0/0.02)
P27134	Carbonic anhydrase	0.45/0.26/0.76 (0.26/0.25/0.62)	0.1/0.28/0.79 (0.07/0.31/0.68)	0.11/0.07/0.66 (0.09/0.24/0.49)
Q31NB2	Ribulose 1,5-bisphosphate carboxylase small subunit	0.03/0.03/0.03 (0/0/0)	0.07/0.06/0.08 (0/0.03/0)	0.07/0.08/0.11 (0/0.04/0)
Q9F1R2	HTH-type transcriptional activator CmpR	0.56/0.35/0.52 (0.62/0.02/0.2)	0.38/0.72/0.45 (0.49/0.12/0.2)	0.22/0.1/0.04 (0.11/0/0.03)
Q55107	Bicarbonate transport ATP-binding protein CmpC	0.03/0.05/0.17 (0.01/0.01/0.01)	0.07/0.03/0.13 (0.05/0.01/0.01)	0.02/0.05/0.06 (0.02/0.05/0)
P39660	Bicarbonate-binding protein CmpA	0.02/0.02/0.02 (0/0/0)	0.03/0.05/0.03 (0/0/0)	0.02/0.01/0.02 (0/0/0)

Q8VPV7	CO2 hydration protein	0.3/0.35/0.15 (0.05/0/0.03)	0.2/0.48/0.27 (0.04/0.02/0.11)	0.03/0.03/0.04 (0/0/0.03)
Q31ME6	NAD(P)H-quinone oxidoreductase subunit H	0.32/0.26/0.28 (0.2/0/0.02)	0.17/0.14/0.13 (0.02/0/0.01)	0.06/0.04/0.07 (0.05/0/0.06)
Q31NJ5	NAD(P)H-quinone oxidoreductase subunit I	0.11/0.11/0.12 (0/0/0)	0.12/0.21/0.07 (0/0/0)	0.09/0.03/0.04 (0.01/0/0)
Q31P07	NAD(P)H-quinone oxidoreductase subunit J	0.21/0.26/0.07 (0.01/0.01/0.16)	0.12/0.26/0.08 (0.01/0.05/0.17)	0.06/0.05/0.14 (0.01/0.05/0.2)
Q31P08	NAD(P)H-quinone oxidoreductase subunit K	0.27/0.56/0.46 (0/0/0.02)	0.15/0.47/0.21 (0.02/0.01/0)	0.06/0.03/0.06 (0/0/0)
Q31LQ7	NAD(P)H-quinone oxidoreductase subunit M	0.06/0.25/0.13 (0.02/0.1/0.02)	0.18/0.33/0.27 (0.15/0.29/0.04)	0.15/0.1/0.17 (0.07/0.12/0.02)
Q31L05	NAD(P)H-quinone oxidoreductase subunit N	0.21/0.18/0.47 (0.21/0.07/0.35)	0.21/0.27/0.17 (0.16/0.5/0.31)	0.03/0.02/0.16 (0.12/0.01/0.3)
P38045	Nitrate transport ATP-binding protein NrtC	3.05/1.25/3.66 (0.07/0.5/0.11)	1.37/1.72/6.19 (0.35/0.4/0.05)	2.25/2.19/6.85 (0.13/0.29/0.05)
P38043	Nitrate transport protein NrtA	16.44/3.98/21.28 (0.04/0.03/0.02)	12.71/4.45/17.06 (0.05/0.03/0.03)	2.78/13.18/10.09 (0.28/0.01/0.09)
Q31PU9	Glutamate synthase (Ferredoxin)	1.92/7.24/4.53 (0.31/0/0)	2/6.61/2.78 (0.28/0.01/0)	1.2/4.83/1.27 (0.53/0.08/0.08)
Q9KHM5	Glutamine synthetase III	2.47/3.37/1.38 (0.25/0.01/0)	0.79/1.51/1.45 (0.95/0.21/0)	3.22/8.63/1.58 (0.16/0/0)
Q31L83	Glutamine synthetase	8.32/11.38/15.7 (0/0.01/0.01)	4.7/9.82/9.2 (0/0.01/0.01)	2.27/8.87/7.38 (0.05/0.03/0.03)
P39661	Ferredoxin--nitrite reductase	1.5/8.87/10.28 (0.42/0.03/0.05)	2.83/11.27/7.24 (0.1/0.03/0.19)	2.42/10.67/9.91 (0.11/0.04/0.1)
Q31QV2	Photosystem I iron-sulfur center	0.85/1.6/1.47 (0.28/0.03/0.58)	2.38/1.94/3.94 (0.38/0.04/0.15)	0.1/0.05/0.25 (0.1/0.1/0.07)
Q31LJ0	Photosystem I P700 chlorophyll a apoprotein A1	2.13/4.17/4.41 (0.02/0.06/0.01)	2.49/7.31/3.77 (0.01/0/0.02)	0.36/0.3/0.47 (0.06/0.19/0.31)
Q31LJ1	Photosystem I P700 chlorophyll a apoprotein A2	4.21/6.14/5.25 (0.01/0.07/0.08)	4.57/10.96/9.38 (0.05/0.04/0.01)	0.43/0.68/0.83 (0.32/0.36/0.54)
Q31PI7	Photosystem I reaction center subunit II	0.82/1.94/3.16 (0.26/0.07/0.89)	4.92/3.19/10.96 (0/0.01/0)	0.77/0.08/0.68 (0.94/0/0.84)
Q31NT9	Photosystem I reaction center subunit III	3.44/2.78/7.18 (0.01/0.02/0.01)	3.66/3.6/10.47 (0.03/0/0)	0.47/0.13/1.42 (0.15/0.07/0.37)
Q31NL7	Photosystem I reaction center subunit IV	2.65/9.46/1.53 (0.2/0.09/0.25)	6.98/16.75/2.96 (0.04/0.03/0.07)	0.06/0.18/0.11 (0.08/0/0.18)
P95822	Photosystem I reaction center subunit XI	7.45/7.11/3.13 (0.03/0.25/0.36)	4.74/10.09/6.79 (0.02/0.2/0.27)	0.9/0.53/0.72 (0.22/0.58/0.65)
Q31M07	Photosystem II 12 kDa extrinsic protein	0.15/1.17/1.34 (0.18/0.83/0.44)	2.33/2.61/6.31 (0.11/0.53/0.06)	0.58/0.12/0.72 (0.67/0.17/0.86)
P11004	Photosystem II CP43 reaction center protein	3.6/1.84/2.68 (0/0.97/0.02)	2.4/1.89/2.54 (0/0.25/0)	0.31/0.41/0.21 (0.02/0/0.57)
P31094	Photosystem II CP47 reaction center protein	3.98/1.42/2.56 (0.01/0.11/0.01)	1.64/1.61/2.17 (0.57/0.16/0.04)	0.39/0.23/0.12 (0.06/0.03/0.18)
P11005	Photosystem II D2 protein	1.43/0.72/3.47 (0.3/0.71/0.11)	1.39/1.11/4.33 (0.35/0.58/0.05)	0.09/0.17/0.22 (0/0.06/0.71)
Q31RE4	Photosystem II lipoprotein Psb27	1.91/2.56/2.29 (0.36/0.23/0.49)	3.28/3.13/4.79 (0.19/0.11/0.09)	0.55/0.19/0.24 (1/0.11/0.13)
P11472	Photosystem II manganese-stabilizing polypeptide	0.23/0.47/0.82 (0.02/0/0.33)	0.59/0.66/1.26 (0.93/0.25/0.01)	0.35/0.24/0.56 (0.02/0/0.78)
P04997	Photosystem II protein D1 2	1.85/1.25/2.58 (0.16/0.58/0.04)	2.21/1.94/2.47 (0.1/0.14/0.07)	0.07/0.09/0.06 (0.01/0.2/0.35)

Q31RG2	Phycobilisome 7.8 kDa linker polypeptide, allophycocyanin-associated, core	6.19/19.77/17.22 (0.05/0.01/0.13)	5.35/19.23/17.7 (0.02/0/0.04)	2.05/8.95/5.25 (0.84/0.06/0.65)
Q31RF9	Phycobilisome core-membrane linker polypeptide	1.26/1.45/2.19 (0.85/0.14/0)	1.21/1.74/2.86 (0.12/0.01/0)	0.08/0.24/0.26 (0/0/0.11)
Q31PD9	Phycobilisome rod linker polypeptide	2.27/3.56/2.19 (0.18/0/0.09)	2.36/4.02/2.42 (0.01/0.01/0.48)	0.34/1.77/0.21 (0.01/0.44/0.35)
Q31LK9	Phycobilisome rod-core linker polypeptide	1.02/0.89/1.58 (0.28/0.14/0.74)	1.24/1.1/2.51 (0.24/0.53/0.73)	0.1/0.31/0.25 (0/0.01/0.06)
P55020	Plastocyanin	16.29/9.91/4.61 (0.22/0.03/0)	16.14/14.19/10.57 (0.14/0.02/0)	12.82/3.87/10.57 (0.22/0.05/0)
Q8KPP3	Cytochrome b559 subunit alpha	2.86/2.31/3.53 (0.3/0.91/0.4)	2.15/2.99/4.25 (0.37/0.48/0.39)	0.22/0.1/0.1 (0.38/0.33/0.48)
Q54711	Cytochrome b6	3.37/11.27/4.06 (0.15/0.03/0.02)	0.95/11.69/5.92 (0.13/0.03/0.02)	0.3/5.65/3.08 (0.69/0.03/0.02)
Q54710	Cytochrome b6-f complex subunit 4	2.23/1.08/2.44 (0.41/0.54/0.38)	2.38/1.02/1.47 (0.58/0.92/0.75)	0.3/0.11/0.15 (0.28/0.14/0.29)
Q31LM9	Cytochrome c-550	2.21/1.85/1.2 (0.15/0.04/0.31)	6.43/3.6/5.65 (0.01/0.01/0)	0.91/0.12/0.59 (0.34/0.02/0.83)
P25935	Cytochrome c6	12.02/23.77/23.12 (0.17/0.16/0.2)	20.51/40.93/29.65 (0.12/0.11/0.15)	1.12/0.44/6.49 (0.53/0.88/0.34)
Q31NV8	Cytochrome f	2.07/0.77/2.09 (0.02/0.77/0.08)	1.51/0.74/1.46 (0.02/0.73/0.25)	0.32/0.1/0.5 (0.01/0/0.44)
Q44112	30 kD rod-rod linker	4.49/6.67/4.17 (0.14/0.02/0.05)	7.94/8.79/8.09 (0/0/0)	0.84/6.61/1.5 (0.74/0/0.42)
Q44114	9 kD rod-rod linker	0.94/3.98/2.25 (0.61/0/0.15)	5.3/8.24/10.38 (0/0/0)	1.1/0.65/1.69 (0.7/0.35/0.66)
Q31RG0	Allophycocyanin alpha chain	0.35/1.8/0.92 (0.02/0.95/0.04)	2.23/3.31/5.25 (0.55/0.22/0.01)	0.74/0.21/1.01 (0.15/0.48/0.45)
Q31RG1	Allophycocyanin, beta subunit	1.24/1.33/2.23 (0.18/0.24/0.66)	1.51/1.39/3.22 (0.95/0.1/0.02)	0.09/0.09/0.32 (0/0.01/0)
P0A2Z8	ATP synthase epsilon chain	13.43/11.27/10.28 (0.01/0/0.01)	12.25/12.13/16 (0.02/0/0)	24.89/4.06/10 (0/0.08/0.02)
Q31RF0	ATP synthase gamma chain	6.03/5.06/2.75 (0.01/0/0)	2.99/2.47/2.25 (0.73/0.02/0.02)	3.56/2.83/1.43 (0.86/0.04/0.38)
Q31RF1	ATP synthase subunit alpha	10.09/15.56/9.82 (0/0/0)	7.05/11.48/5.55 (0/0/0)	3.13/8.09/1.84 (0.34/0.01/0.01)
Q31RF3	ATP synthase subunit b	4.74/10.19/1.66 (0.04/0.01/0.11)	3.7/8.71/4.21 (0.04/0.04/0.06)	3.13/4.06/4.06 (0.03/0.2/0.05)
Q31RF4	ATP synthase subunit b'	12.36/21.48/9.29 (0.01/0/0.02)	8.55/16.44/11.27 (0.04/0/0.01)	5.01/12.59/9.29 (0.22/0/0.01)
Q31KS4	ATP synthase subunit beta	7.18/9.91/12.36 (0/0/0)	4.53/6.03/8.63 (0/0/0)	2.23/4.29/4.66 (0/0/0.01)
Q31RF5	ATP synthase subunit c	1.5/12.82/0.16 (0.4/0.36/0.53)	1.17/6.03/1.56 (0.53/0.88/0.85)	1.75/14.45/3.31 (0.34/0.33/0.36)
Q935X4	NADPH-protochlorophyllide oxidoreductase	3.08/8.32/11.8 (0.26/0.02/0.1)	5.15/13.55/13.06 (0/0/0.06)	0.41/1.75/1.79 (0.05/0.82/0.22)
Q31PM2	Porphobilinogen deaminase	6.37/20.32/22.28 (0.11/0/0.03)	12.59/25.35/23.33 (0.05/0/0.03)	15.14/13.18/20.32 (0.04/0.01/0.02)
P16891	Uroporphyrinogen decarboxylase	12.94/14.06/7.66 (0.06/0.05/0.11)	3.8/13.18/7.11 (0.02/0.05/0.14)	3.77/8.09/7.73 (0.46/0.08/0.47)
Q31QQ4	Mg-protoporphyrin IX chelatase	6.55/7.73/12.82 (0/0/0.1)	3.4/7.87/8.79 (0.01/0/0.06)	4.92/6.08/5.2 (0/0/0.13)

Q31LY2	Magnesium-protoporphyrin IX monomethyl ester [oxidative] cyclase	2.51/2.68/3.53 (0.13/0.3/0.14)	0.38/2.86/3.56 (0.17/0.15/0.22)	0.7/0.35/0.5 (0.73/0.63/0.51)
Q9Z3G6	Heme oxygenase (Decyclizing)	5.35/2.73/2.68 (0.2/0.07/0.43)	5.15/2.44/2.99 (0.65/0.53/0.32)	4.29/2.05/2.91 (0.08/0.43/0.33)
Q31LA2	Hydrogenobyrinic acid a,c-diamide cobaltochelatase	10.09/6.61/4.7 (0/0.36/0.45)	7.59/12.71/10.47 (0/0.37/0.07)	2.09/8.63/5.06 (0/0.49/0.46)
Q31QJ2	Glutamate-1-semialdehyde 2,1-aminomutase	4.06/15.42/10.57 (0/0/0)	5.35/12.25/12.25 (0.02/0/0)	1.84/10.86/6.98 (0.02/0/0.02)
Q31RA2	Geranylgeranyl reductase	2.17/1.74/3.08 (0.25/0.08/0.01)	0.68/1.21/2.27 (0.99/0.29/0.08)	0.13/0.15/0.34 (0.04/0.04/0.95)
Q31M79	Flavoprotein	0.29/0.16/0.3 (0.2/0.04/0.17)	0.13/0.23/0.11 (0.14/0.04/0.01)	0.2/0.13/0.12 (0.15/0.04/0.1)
Q31NJ4	NADH dehydrogenase subunit 6	0.5/0.19/0.79 (0.03/0.12/0.02)	0.26/0.11/0.65 (0.05/0.13/0.02)	0.17/0.05/0.31 (0.02/0.05/0)
Q31PZ6	NADPH-glutathione reductase	0.34/0.86/0.34 (0.23/0.57/0.17)	0.26/0.64/0.26 (0.18/0.96/0.19)	0.22/0.79/0.53 (0.16/0.62/0.42)
Q31KG4	Chaperon-like protein for quinone binding in photosystem II	0.84/0.5/0.5 (0.75/0.22/0.3)	0.5/0.45/0.53 (0.33/0.17/0.32)	0.49/0.21/0.29 (0.32/0.01/0.23)
Q31QD9	Probable ferredoxin	0.14/0.38/0.29 (0.04/0.28/0.19)	0.08/0.94/0.47 (0.03/0.5/0.27)	0.74/0.5/0.86 (0.33/0.4/0.66)
P18655	Superoxide dismutase [Fe]	0.11/0.19/0.25 (0.04/0.02/0.25)	0.11/0.08/0.37 (0.23/0/0.07)	1.28/0.22/0.99 (0.57/0.01/0.43)
P12243	Thioredoxin 1	0.23/1.32/0.58 (0.09/0.53/0.32)	2.33/3.19/4.09 (0.01/0/0)	1/0.16/1.15 (0.4/0.76/0.1)
Q7X4K8	Thioredoxin peroxidase	0.25/0.25/0.3 (0/0.01/0.06)	0.07/0.45/0.24 (0/0.02/0.22)	0.21/0.07/0.44 (0/0/0.13)
Q31MK3	Thiosulphate-binding protein	0.23/0.26/0.33 (0.25/0.01/0.29)	0.18/0.06/0.3 (0.22/0.02/0.04)	0.21/0.08/0.09 (0.26/0/0.08)
Q79PF2	Glutathione peroxidase	0.46/0.17/0.14 (0.3/0.14/0.21)	0.77/0.14/0.36 (0.63/0.12/0.32)	0.52/0.67/1.28 (0.34/0.5/0.66)
Q31Q49	Glutathione S-transferase	0.12/0.11/0.28 (0.01/0.01/0)	0.35/0.08/0.16 (0.15/0/0.01)	0.15/0.08/0.35 (0.21/0.01/0.05)
Q31QJ5	Bacterioferritin comigratory protein	0.28/0.09/0.04 (0.1/0.2/0.04)	0.64/0.06/0.34 (0.36/0.19/0.14)	0.6/0.13/0.48 (0.34/0.19/0.23)
Q31L59	Bacterioferritin comigratory protein	0.3/0.63/0.11 (0.04/0.65/0)	0.37/0.39/0.35 (0/0.29/0.44)	1.28/0.3/1.01 (0.76/0.1/0.89)
Q31KE0	1-Cys peroxiredoxin	0.14/0.62/0.22 (0.04/0.94/0.14)	0.61/0.79/0.74 (0.18/0.47/0.89)	0.29/0.05/0.36 (0.01/0/0.06)
P22880	10 kDa chaperonin	2.96/14.72/4.83 (0.06/0/0.6)	7.05/16.14/7.73 (0.02/0/0.36)	13.06/8.79/9.73 (0.01/0/0.11)
P22879	60 kDa chaperonin	3.37/6.37/5.06 (0.02/0/0)	2.94/4.53/5.5 (0.04/0/0)	4.45/5.45/5.86 (0/0/0)
Q31QF2	60 kDa chaperonin	1.69/2.73/2.54 (0.13/0.01/0.05)	0.51/0.52/1.15 (0.95/0.02/0.34)	1.47/3.56/3.4 (0.13/0/0.01)
Q31P64	Putative CheA signal transduction histidine kinases, no good domain identification	3.91/2.68/1.39 (0.18/0.53/0.39)	6.08/5.55/1.61 (0.14/0.04/0.35)	5.06/2.51/1.33 (0.15/0.25/0.38)
Q31NQ0	Putative modulator of DNA gyrase	0.13/0.78/0.8 (0.16/0.62/0.78)	0.32/0.18/0.46 (0.16/0.36/0.26)	0.12/0.39/0.88 (0.14/0.25/0.75)
P27477	Putative thiosulfate sulfurtransferase	18.88/19.77/6.25 (0.06/0.17/0.28)	16.75/34.04/17.7 (0.01/0.13/0.19)	15.85/13.55/8.47 (0.04/0.22/0.34)

Q31MF5	Ferredoxin-thioredoxin reductase, catalytic chain	1.49/10.09/1.29 (0.12/0.18/0.81)	1.89/20.7/1.85 (0.08/0.13/0.39)	1.12/7.05/2.49 (0.5/0.2/0.26)
Q8GIT7	DNA gyrase subunit B	9.12/0.52/0.65 (0.3/0.38/0.2)	0.28/0.33/0.34 (0.82/0.03/0.02)	10.09/0.74/0.3 (0.28/0.96/0.13)
Q31LB8	Anti-sigma factor antagonist	2.47/7.94/2.56 (0.23/0.08/0.05)	4.33/10.86/2.38 (0.16/0.02/0.44)	3.56/4.45/2.25 (0.16/0.29/0.15)
Q59984	Protein GrpE	0.44/0.3/0.49 (0.08/0.22/0.4)	0.52/0.55/0.47 (0.78/0.81/0.37)	0.68/0.41/1.16 (0.43/0.17/0.82)
P54415	ATP-dependent Clp protease proteolytic subunit 1	0.56/0.71/0.87 (0.15/0.65/0.56)	0.1/0.37/0.61 (0.06/0.37/0.01)	0.32/0.84/0.81 (0.11/0.75/0.07)
Q9L4P4	Putative ATP-dependent Clp protease proteolytic subunit-like	0.32/0.27/0.53 (0.11/0.17/0.03)	0.14/0.18/0.28 (0.07/0.05/0.01)	0.5/0.91/0.36 (0.09/1/0.19)
Q31QY2	D-fructose 1,6-bisphosphatase class 2/sedoheptulose 1,7-bisphosphatase	1.94/3.28/2.31 (0.25/0.03/0.34)	2.63/3.6/2.38 (0.02/0/0.02)	0.42/1.13/0.32 (0.43/0.31/0.15)
Q9R6W2	Glyceraldehyde-3-phosphate dehydrogenase	7.66/6.08/3.63 (0.04/0.01/0.08)	5.86/4.06/3.66 (0.05/0.01/0.09)	5.3/1.39/3.77 (0.17/0.96/0.22)
Q31QU9	Transketolase	4.79/3.05/8.09 (0.03/0.01/0)	2.56/2.36/5.01 (0.63/0.11/0.01)	1.54/4.21/1.98 (0.37/0.06/0.8)
Q31MH0	Isocitrate dehydrogenase [NADP]	0.48/0.6/1.07 (0.72/0.22/0.18)	0.21/0.21/0.34 (0.01/0/0.58)	0.24/0.55/0.24 (0.13/0/0.66)
Q31PT6	Aconitate hydratase B	0.38/0.58/0.63 (0.11/0.85/0.42)	0.41/0.22/0.13 (0.04/0.08/0.03)	0.27/0.82/0.77 (0.03/0.67/0.61)
Q31R18	2,3-bisphosphoglycerate-independent phosphoglycerate mutase	2.54/4.66/4.41 (0.25/0.08/0.37)	3.7/9.04/4.21 (0.18/0.04/0.39)	3.84/7.11/6.03 (0.18/0.08/0.26)
Q31S39	Pyruvate kinase	1.89/2.33/3.31 (0.14/0.09/0)	1.36/1.89/2.54 (0.85/0.55/0)	1.34/3.13/2 (0.88/0.02/0.02)
Q31P73	Phosphoglycerate kinase	3.47/4.92/6.98 (0/0/0)	3.13/5.97/6.25 (0/0/0)	0.84/2.7/1.41 (0.67/0.06/0.23)
Q31RZ4	Pyruvate/2-oxoglutarate dehydrogenase complex dehydrogenase (E1) component	4.88/4.25/3.31 (0.01/0.17/0.13)	2.65/3.91/3.1 (0.01/0.11/0.14)	0.87/3.66/0.91 (0.77/0.03/0.3)
Q31PC1	Dihydrolipoamide acetyltransferase component of pyruvate dehydrogenase complex	4.09/2.88/2.49 (0.19/0.1/0.08)	0.6/3.66/2.68 (0.69/0.09/0.05)	0.73/1.26/1.16 (0.39/0.2/0.89)
Q31NZ1	Dihydrolipoyl dehydrogenase	2.25/5.97/2.13 (0.45/0/0.07)	2.63/2.54/1.6 (0.28/0.14/0.02)	6.85/2.31/3.02 (0.05/0.1/0)
Q31NI7	Acetyl-coenzyme A synthetase	1.82/4.92/8.47 (0.28/0.01/0.01)	0.95/4.33/8.02 (0.57/0.07/0.02)	0.37/2.47/1.58 (0.83/0.03/0.58)
P21577	6-phosphogluconate dehydrogenase, decarboxylating	0.12/0.06/0.17 (0.01/0/0)	0.06/0.08/0.1 (0/0/0)	0.16/0.13/0.19 (0.07/0.05/0)
Q31KU2	Transaldolase	0.29/0.18/0.27 (0.09/0/0.02)	0.19/0.19/0.29 (0.09/0/0.02)	0.45/0.53/0.77 (0.25/0.3/0.2)
P29686	Glucose-6-phosphate 1-dehydrogenase	0.33/0.21/0.19 (0.19/0.04/0.04)	0.15/0.11/0.1 (0.1/0.02/0)	1.49/0.8/0.7 (0.47/0.61/0.35)
Q31QF3	3-oxoacyl-[acyl-carrier-protein] reductase	0.68/1.67/0.77 (0.19/0.37/0.08)	0.59/0.95/0.86 (0.22/0.86/0.07)	0.36/0.62/0.19 (0.13/0.33/0.26)
Q31QV0	3-oxoacyl-[acyl-carrier-protein] synthase 2	3.16/10.38/2.75 (0.08/0.01/0.32)	6.79/11.38/1.71 (0.05/0.01/0.4)	6.73/13.93/4.06 (0.08/0.05/0.2)
Q31N38	D-3-phosphoglycerate dehydrogenase	1.71/2.23/2.13 (0.25/0.12/0.28)	0.48/0.85/0.86 (0.08/0.75/0.73)	0.69/1.46/0.4 (0.67/0.95/0.56)

P27366	Sulfate-binding protein	0.02/0.07/0.16 (0.03/0.02/0.19)	0.14/0.04/0.08 (0.02/0.02/0.3)	0.13/0.06/0.06 (0.02/0.01/0.07)
Q5N0R0	Iron deficiency-induced protein A	0.14/0.19/0.7 (0.01/0.02/0.87)	0.08/0.04/0.16 (0.06/0.01/0.22)	0.05/0.09/0.07 (0.04/0/0.23)
Q31ND0	Iron transport system substrate-binding protein	0.16/0.07/0.21 (0/0/0)	0.16/0.04/0.08 (0/0/0)	0.05/0.03/0.04 (0/0/0)
Q31KE5	Phosphate binding protein	1.72/7.11/10.86 (0.34/0.01/0.22)	1.13/2.56/4.92 (0.66/0.3/0.47)	1.58/6.43/18.2 (0.38/0.91/0.16)
Q31NM5	ATP-dependent zinc metalloprotease FtsH	0.19/0.14/0.3 (0/0/0.16)	0.17/0.1/0.19 (0.01/0/0.15)	0.18/0.48/0.54 (0.03/0.03/0.42)
Q31QC5	C-terminal processing peptidase-2. Serine peptidase. MEROPS family S41A	0.21/0.12/0.33 (0.87/0.08/0.43)	0.54/0.41/0.42 (0.68/0.02/0.67)	5.65/4.45/4.97 (0.19/0/0)
Q31LX3	Signal peptide peptidase A. Serine peptidase. MEROPS family S49	1.32/0.39/0.29 (0.51/0.27/0.5)	0.22/0.21/0.41 (0.51/0.04/0.43)	0.14/0.17/0.09 (0.33/0.02/0.21)
Q31RW6	Cysteine synthase	0.32/0.23/0.75 (0.17/0.41/0.36)	0.2/0.6/0.37 (0.13/0.59/0.05)	0.25/0.14/0.26 (0.09/0.02/0.08)
Q31QL1	Dihydroxy-acid dehydratase	2.17/0.92/1.87 (0.21/0.23/0.08)	0.83/0.31/0.5 (0.81/0.02/0.9)	0.55/0.15/0.27 (0.47/0.01/0.17)
P29820	Peptidyl-prolyl cis-trans isomerase	0.12/0.45/0.18 (0.02/0.55/0.1)	0.49/0.79/0.44 (0.14/0.6/0.19)	0.7/0.14/1.16 (0.42/0.51/0.76)
Q31KN5	Peptidyl-prolyl cis-trans isomerase	1.16/1.46/1.21 (0.76/0.14/0.63)	1.94/2.33/3.02 (0.62/0.06/0.09)	3.28/0.7/1.47 (0.72/0.26/0.32)
Q31NT0	Arsenite-activated ATPase (ArsA)	0.54/0.19/0.17 (0.12/0/0)	0.16/0.28/0.16 (0.03/0/0)	0.23/0.52/0.31 (0.11/0.03/0.01)
Q31LG8	ATPase	0.19/0.1/0.41 (0.05/0.08/0.49)	0.27/0.13/0.39 (0.03/0.21/0.51)	0.15/0.52/0.13 (0.04/0.13/0.27)
Q31R70	ATPase	0.16/0.07/0.11 (0.07/0.19/0.16)	0.39/0.2/0.22 (0.22/0.39/0.17)	0.16/0.61/1.21 (0.05/0.66/0.93)
Q31PM9	ATPase	0.69/0.51/0.62 (0.6/0.04/0.55)	0.06/0.11/0.48 (0.19/0.02/0.35)	0.39/0.65/1.05 (0.38/0.67/0.96)
P63200	30S ribosomal protein S12	0.64/0.52/0.42 (0.98/0.59/0.26)	0.11/0.18/0.17 (0.07/0.81/0.25)	0.64/0.95/0.67 (0.48/0.22/0.69)
Q31L28	30S ribosomal protein S13	4.41/3.94/3.87 (0.33/0.1/0.37)	3.47/2.83/1.21 (0.38/0.28/0.84)	4.33/1.36/2 (0.13/0.04/0.59)
Q31KU0	30S ribosomal protein S15	3.05/8.39/5.55 (0.27/0.11/0.24)	0.46/9.46/4.21 (0.3/0.11/0.39)	0.39/3.6/3.87 (0.38/0.24/0.4)
Q31MB7	30S ribosomal protein S16	3.34/5.15/3.73 (0.07/0.02/0.17)	3.98/3.53/3.4 (0.07/0.06/0.08)	2.96/3.22/2.7 (0.82/0.05/0.02)
Q31P66	30S ribosomal protein S18	2.88/10.19/4.74 (0.06/0/0.03)	1.85/4.83/1.43 (0.21/0.08/0.25)	2.73/4.37/3.63 (0.01/0/0.01)
Q31L11	30S ribosomal protein S19	0.61/1.11/1.33 (0.21/0.83/0.66)	0.4/0.15/0.16 (0.16/0.46/0.58)	1.91/1.41/1.54 (0.43/0.58/0.31)
Q31K59	30S ribosomal protein S2	2.54/3.05/4.25 (0.02/0.48/0.03)	1.53/2.63/2.33 (0.3/0.27/0.19)	0.21/1.17/0.38 (0.19/0.76/0.85)
Q31L13	30S ribosomal protein S3	3.7/7.87/6.61 (0.02/0/0)	1.29/5.35/3.5 (0.12/0.01/0)	1.6/3.84/1.71 (0.18/0/0.1)
Q31N52	30S ribosomal protein S4	2.49/4.21/2.15 (0.03/0.17/0.77)	1.22/3.05/1.36 (0.5/0.22/0.88)	1.89/1.82/0.36 (0.32/0.57/0.27)
Q31L23	30S ribosomal protein S5	2.4/3.47/3.66 (0.53/0.17/0.46)	1.14/1.6/2.01 (0.66/0.69/0.87)	2.63/3.37/8.02 (0.12/0.3/0.01)
Q31PV3	30S ribosomal protein S7	7.87/10.28/13.43 (0.02/0/0.08)	13.55/8.71/12.25 (0.03/0.05/0.06)	1.71/8.39/4.25 (0.43/0.06/0.17)

Q31L20	30S ribosomal protein S8	4.29/3.56/5.97 (0.12/0.06/0.19)	2.15/1.6/7.24 (0.38/0.14/0)	3.73/3.84/4.13 (0.08/0.07/0.28)
Q31QK4	50S ribosomal protein L1	6.03/6.92/6.37 (0.51/0.01/0.04)	5.11/5.7/5.97 (0.38/0.01/0.04)	9.04/5.55/9.2 (0.09/0.04/0.03)
Q31QK5	50S ribosomal protein L10	3.22/7.66/3.53 (0.07/0/0.16)	3.7/7.52/4.88 (0.06/0/0.09)	3.19/3.34/8.24 (0.04/0.09/0.16)
Q31QK3	50S ribosomal protein L11	4.33/10.38/14.45 (0.26/0/0)	3.91/7.8/18.03 (0.17/0/0)	5.81/2.78/14.32 (0.24/0.02/0)
Q31L33	50S ribosomal protein L13	12.47/9.46/7.31 (0.02/0.02/0.04)	18.2/7.94/9.55 (0/0.11/0.01)	9.64/6.37/8.47 (0.04/0.19/0.1)
Q31L17	50S ribosomal protein L14	9.29/6.49/9.12 (0.02/0/0.65)	6.61/6.73/10.67 (0/0.12/0.21)	4.29/2.61/7.11 (0.11/0.49/0.74)
Q31L31	50S ribosomal protein L17	4.41/6.98/3.4 (0.1/0.1/0.46)	6.73/5.5/3.66 (0.05/0.15/0.03)	4.25/3.44/1.63 (0.11/0.16/0.82)
Q31K48	50S ribosomal protein L19	1.61/9.91/8.55 (0.2/0/0.41)	2.54/6.67/16.29 (0.05/0/0.02)	2.42/4.13/13.18 (0.37/0/0.1)
Q31NR2	50S ribosomal protein L20	14.45/11.07/3.34 (0.02/0.31/0.06)	14.59/7.87/6.73 (0.01/0.25/0.02)	0.22/7.38/0.09 (0.8/0.38/0.11)
Q9Z3H5	50S ribosomal protein L21	1.74/16.9/2.51 (0.19/0.18/0.32)	2.19/19.05/3.77 (0.13/0.04/0.25)	3.28/11.8/4.17 (0.03/0.11/0.14)
Q31L12	50S ribosomal protein L22	2.29/17.06/4.88 (0.36/0.01/0.02)	1.87/13.18/2.86 (0.39/0.02/0.2)	1.87/12.25/1.26 (0.62/0.09/0.64)
Q31L09	50S ribosomal protein L23	8.24/4.61/3.63 (0.02/0.02/0.22)	6.79/4.41/4.33 (0.02/0.03/0.07)	9.64/2.21/9.64 (0.08/0.02/0.01)
Q31L18	50S ribosomal protein L24	1.66/4.61/1.72 (0.23/0.03/0.2)	1.2/1.6/0.7 (0.54/0.24/0.73)	1.42/2.49/2.96 (0.13/0.02/0)
Q31S95	50S ribosomal protein L28	6.67/1.94/2.07 (0.25/0.15/0.22)	2.78/0.82/2.21 (0.52/0.86/0.16)	3.25/1.66/0.47 (0.27/0.91/0.61)
Q31L15	50S ribosomal protein L29	4.45/6.92/8.39 (0.08/0.01/0.34)	5.86/9.55/17.06 (0.25/0/0.27)	10.57/5.06/25.82 (0/0.05/0.23)
Q31L07	50S ribosomal protein L3	2.7/7.94/4.7 (0.19/0.23/0.01)	1.24/7.87/4.13 (0.93/0.15/0.05)	0.82/1.75/3.37 (0.62/0.88/0.02)
Q31L35	50S ribosomal protein L31	7.18/13.8/3.84 (0.09/0.06/0.36)	4.13/17.38/20.32 (0.15/0.03/0.28)	12.02/5.45/14.32 (0.07/0.33/0.42)
Q31L08	50S ribosomal protein L4	6.43/10.67/7.8 (0.03/0.12/0.18)	3.4/9.55/10.86 (0.04/0.23/0.16)	3.4/3.16/2.94 (0.1/0.24/0.28)
Q31L19	50S ribosomal protein L5	6.49/4.92/9.64 (0/0.18/0.08)	8.24/5.92/8.47 (0/0.02/0.08)	5.6/1.5/4.66 (0.01/0.99/0.14)
Q31L21	50S ribosomal protein L6	1.58/3.31/6.49 (0.71/0.15/0.02)	1.85/2.47/2.78 (0.16/0.1/0.04)	2.07/2.03/4.21 (0.38/0.17/0.03)
Q31QK6	50S ribosomal protein L7/L12	11.8/10.09/17.86 (0/0.01/0)	10.86/10.19/18.2 (0/0.02/0)	9.82/1.45/11.07 (0/0.14/0)
Q31K30	50S ribosomal protein L9	0.74/4.74/1.85 (0.23/0.02/0.56)	1.56/2.65/1.54 (0.65/0.13/0.53)	1.79/3.98/6.37 (0.67/0.06/0.02)
O05161	Probable 30S ribosomal protein PSRP-3	4.92/9.46/1.92 (0/0.09/0.33)	6.03/12.02/3.34 (0.11/0.07/0.06)	3.98/2.61/3.7 (0.05/0.18/0.96)
Q31QT3	Ribosome-binding ATPase YchF	2.75/9.55/5.5 (0.23/0.05/0.17)	2.56/8.02/2.38 (0.25/0.03/0.26)	1.22/6.19/11.38 (0.68/0.13/0.03)
Q31QY0	Ribosome-recycling factor	5.97/11.91/2.58 (0.04/0/0.08)	13.68/19.41/9.38 (0.02/0/0.04)	20.51/8.02/9.91 (0.02/0/0.04)
Q31QE3	SSU ribosomal protein S1P	4.83/7.59/8.71 (0.04/0/0)	2.13/4.49/10.09 (0.1/0/0)	1.27/2.42/8.39 (0.33/0/0)
Q31PV4	Elongation factor G	3.73/2.25/10.19 (0/0.01/0)	1.43/1.5/5.97 (0.59/0.23/0)	2.33/2.47/5.5 (0.09/0/0)

Q54760	Elongation factor P	3.37/11.48/7.94 (0.44/0.01/0.59)	4.61/7.45/15.28 (0.26/0.01/0.06)	5.45/5.4/12.82 (0.81/0.18/0.23)
Q31K58	Elongation factor Ts	5.6/8.32/10.47 (0.13/0/0)	4.21/9.64/11.17 (0.07/0/0)	6.43/3.37/8.09 (0.06/0/0)
P32084	Uncharacterized HIT-like protein Synpcc7942_1390	0.36/0.11/0.07 (0.42/0.25/0.23)	0.41/0.13/0.43 (0.33/0.3/0.72)	1.03/0.21/0.68 (0.71/0.36/0.98)
Q31KY2	Uncharacterized protein	0.06/0.21/0.16 (0/0.01/0)	0.18/0.51/0.12 (0.15/0.02/0)	0.07/0.09/0.22 (0.02/0.01/0.08)
Q31KJ4	Uncharacterized protein	0.26/0.08/0.2 (0.05/0.03/0.02)	0.08/0.03/0.03 (0.09/0.03/0.01)	0.27/0.35/0.12 (0.18/0.08/0.01)
Q31LY1	Uncharacterized protein	0.17/0.4/0.53 (0.03/0.07/0.39)	0.28/0.65/0.82 (0.07/0.13/0.99)	0.07/0.05/0.66 (0.01/0.01/0.36)
Q8GIS3	Uncharacterized protein	0.32/0.1/0.35 (0.21/0.03/0.15)	0.65/0.1/0.27 (0.47/0.03/0.13)	0.05/0.52/0.39 (0.08/0.15/0.07)
Q31QW7	Uncharacterized protein	0.59/1.09/0.62 (0.41/0.9/0.32)	0.88/0.72/0.74 (0.81/0.16/0.54)	0.66/0.1/0.26 (0.49/0.19/0.03)
Q31PX2	Uncharacterized protein	0.16/0.21/0.39 (0.21/0.06/0.34)	0.13/0.23/0.45 (0.37/0.08/0.29)	0.39/0.2/0.54 (0.29/0.06/0.4)
Q54759	Uncharacterized protein	0.13/0.22/0.27 (0/0.12/0.05)	0.26/0.72/0.24 (0/0.48/0.09)	0.37/0.33/0.58 (0.19/0.07/0.32)
Q31RI3	Uncharacterized protein	0.06/0.35/0.27 (0.01/0.02/0.18)	0.13/0.56/0.26 (0.02/0.09/0.18)	0.53/0.58/0.39 (0.16/0.09/0.25)
Q31M27	Uncharacterized protein	0.16/0.23/0.2 (0.85/0.04/0.19)	0.69/0.98/1.61 (0.45/0.6/0.3)	5.3/2.42/6.31 (0.08/0/0)
Q8GMR4	Uncharacterized protein	0.25/0.67/0.77 (0.03/0.07/0.63)	0.03/0.03/0.06 (0.04/0.01/0.01)	1.03/1.32/2.21 (0.87/0.32/0.08)
Q31KK9	Uncharacterized protein	0.13/0.2/0.06 (0.01/0.01/0)	0.32/0.18/0.15 (0.05/0/0.02)	0.94/0.45/0.84 (0.79/0.08/0.33)
Q31PS0	Uncharacterized protein	0.41/0.36/0.16 (0.08/0.47/0.15)	0.17/0.36/0.43 (0.07/0.5/0.68)	0.69/0.19/0.92 (0.84/0.1/0.81)
Q31L98	Uncharacterized protein	0.39/0.65/0.59 (0.25/0.48/0.96)	0.39/0.51/0.38 (0.24/0.34/0.58)	1.01/0.37/0.74 (0.88/0.24/0.72)
Q31RP7	Uncharacterized protein	0.56/1.08/2.11 (0.26/0.76/0.4)	2.15/2.01/4.61 (0.07/0.19/0.02)	0.32/0.28/1.04 (0.02/0.15/0.69)
Q31RH4	Uncharacterized protein	0.46/0.44/5.86 (0.58/0.27/0.14)	2.78/1.87/4.66 (0.18/0.74/0.16)	1.6/0.16/2.38 (0.27/0.35/0.27)
Q31PZ5	Uncharacterized protein	0.06/0.33/0.12 (0.1/0.23/0.43)	0.51/0.95/0.72 (0.03/0.93/0.65)	0.82/0.14/0.98 (0.29/0.06/0.16)
Q8GA99	Uncharacterized protein	0.39/0.58/0.34 (0.13/0.75/0.07)	0.53/1.24/0.89 (0.29/0.86/0.3)	0.55/0.39/0.89 (0.35/0.99/0.1)
Q31PM5	Uncharacterized protein	0.77/0.16/0.1 (0.62/0.12/0.3)	1.49/1.24/1.1 (0.43/0.62/0.55)	2.56/1.61/3.87 (0.23/0.55/0.26)
Q31PK1	Uncharacterized protein	4.53/24.89/13.8 (0.16/0.15/0.2)	2.63/27.54/17.38 (0.24/0.15/0.16)	1.67/33.73/8.24 (0.41/0.14/0.79)
Q31LN3	Uncharacterized protein	0.79/0.13/0.89 (0.66/0.12/0.79)	0.1/0.16/0.37 (0.11/0.26/0.83)	0.54/0.93/0.69 (0.37/0.44/0.72)
Q31LJ5	Uncharacterized protein	1.01/0.29/0.79 (0.98/0.26/0.43)	0.39/0.41/0.16 (0.26/0.21/0.06)	0.67/0.48/0.4 (0.5/0.37/0.51)
Q31KI1	Uncharacterized protein	12.47/1.18/1.36 (0.03/0.41/0.09)	17.06/1.98/1.57 (0.03/0.23/0.08)	24.89/1.02/1.53 (0.03/0.55/0.07)
Q8KPQ8	Uncharacterized protein SEE0036	0.07/0.45/0.09 (0/0/0.02)	0.21/0.59/0.28 (0/0/0.16)	0.25/0.16/0.51 (0.01/0/0.03)
Q31K01	3-octaprenyl-4hydroxybenzoate decarboxylase	0.27/0.22/0.28 (0.04/0/0.06)	0.38/0.09/0.07 (0.15/0/0)	0.51/0.38/0.14 (0.08/0.17/0.01)

Q31QC4	4-hydroxy-3-methylbut-2-en-1-yl diphosphate synthase (ferredoxin)	1.14/1.22/1.45 (0.46/0.44/0.24)	0.72/0.44/0.91 (0.1/0.22/0.82)	0.65/0.08/0.31 (0.05/0/0.45)
Q31LH6	5'-nucleotidase SurE	0.41/0.24/0.49 (0.26/0.02/0.33)	0.56/0.26/0.29 (0.38/0.21/0.2)	0.58/0.19/0.61 (0.4/0.03/0.43)
Q31RZ8	Acetolactate synthase	2.7/5.55/1.79 (0.23/0/0.27)	1.79/4.53/1.58 (0.39/0.02/0.32)	1.17/1.8/1.11 (0.37/0.61/0.98)
Q31KD5	Adenine phosphoribosyltransferase	0.47/0.12/0.34 (0.5/0.06/0.29)	0.26/0.11/0.35 (0.5/0.12/0.26)	0.91/0.57/0.9 (0.71/0.36/0.69)
Q31QL9	Adenosylhomocysteinase	16.6/11.07/8.39 (0/0/0.03)	15.7/9.91/6.98 (0/0/0.08)	6.85/11.69/8.63 (0.02/0/0.13)
Q31KW8	ADP-ribose pyrophosphatase	1.57/0.08/0.24 (0.45/0.01/0.23)	0.64/0.15/0.15 (0.47/0.43/0.22)	0.74/0.23/0.58 (0.59/0.08/0.5)
Q31L79	Alanine-glyoxylate aminotransferase	2.94/19.59/16 (0.08/0.01/0.05)	3.5/19.95/13.06 (0.07/0.01/0.07)	3.31/9.46/4.7 (0.07/0.05/0.14)
Q55038	Amidophosphoribosyltransferase	0.16/0.44/0.95 (0.02/0.04/0.92)	0.07/0.11/0.9 (0.01/0/0.82)	0.08/0.11/0.5 (0.01/0/0.24)
Q31KZ1	Bacterial nucleoid protein Hbs	0.12/0.29/0.04 (0.03/0/0.03)	0.41/0.86/0.57 (0/0.24/0.16)	0.57/0.08/0.56 (0.36/0.01/0.14)
Q31P81	Bacterial nucleoid protein Hbs	2.03/6.31/2.75 (0.04/0.39/0.2)	7.73/13.68/9.38 (0/0.04/0)	2.36/1.21/0.82 (0.2/0.42/0.47)
P52023	Beta sliding clamp	1.57/1.94/1.85 (0.35/0.09/0.29)	1.84/2.19/1.74 (0.41/0.06/0.24)	1.66/1.2/0.53 (0.51/0.47/0.39)
Q8VPV6	Beta-Ig-H3/fasciclin	0.09/0.19/0.15 (0.1/0.07/0.16)	0.14/0.22/0.2 (0.18/0.06/0.18)	0.01/0.01/0.01 (0.03/0/0.04)
Q31Q60	Bifunctional protein FolD	0.28/0.45/0.13 (0.11/0.33/0.6)	0.37/0.21/0.64 (0.14/0.1/0.8)	0.52/0.38/0.34 (0.27/0.31/0.47)
Q31PH5	CheA signal transduction histidine kinase	3.02/1.21/2.99 (0.15/0.21/0.03)	3.37/1.82/4.29 (0.14/0.12/0.02)	2.54/1.8/3.08 (0.17/0.1/0.05)
Q31RS5	Chorismate synthase	0.36/0.25/1.02 (0.05/0.21/0.95)	0.51/0.22/0.63 (0.55/0.24/0.45)	0.21/0.67/1.08 (0.29/0.62/0.86)
Q79PF4	Circadian clock protein kinase KaiC	0.45/0.24/0.34 (0.24/0.1/0.01)	0.76/0.29/0.75 (0.73/0.28/0.17)	0.4/1.26/0.38 (0.09/0.99/0.14)
Q6BBK3	CP12	2.01/3.91/8.71 (0/0.68/0.09)	12.59/6.31/15.28 (0/0.14/0.03)	8.79/0.69/12.94 (0/0.71/0.03)
Q935X8	DhnA	0.79/0.4/0.42 (0.67/0.05/0.27)	0.7/0.28/0.36 (0.54/0.03/0.23)	0.6/0.52/0.64 (0.42/0.08/0.47)
Q31QZ9	Dihydroorotase	0.52/0.45/0.21 (0.3/0.21/0.01)	0.26/0.55/0.28 (0.21/0.47/0)	1.6/0.44/0.6 (0.52/0.46/0.04)
Q31Q61	Farnesyl-diphosphate synthase	1.21/12.82/12.94 (0.54/0.01/0.05)	2.91/12.47/13.43 (0.06/0.02/0.01)	2.11/20.89/11.91 (0.11/0.01/0.14)
Q31KD9	General secretion pathway protein D	1.56/1.42/1.42 (0.91/0.53/0)	0.19/3.87/1.57 (0.44/0.18/0)	2.75/5.2/3.94 (0.49/0.15/0)
Q31RU8	GMP synthase [glutamine-hydrolyzing]	0.82/1.49/3.56 (0.93/0.13/0.23)	0.69/1.53/0.3 (0.72/0.2/0.28)	2.21/5.75/4.41 (0.34/0/0.15)
Q54769	GTP cyclohydrolase 1	0.45/0.92/0.63 (0.32/0.55/0.58)	0.44/0.97/0.5 (0.29/0.15/0.19)	0.6/0.61/0.62 (0.42/0.01/0.55)
Q31N14	GTP-binding protein TypA	0.61/1.27/1.06 (0.87/0.45/0.23)	0.25/0.39/0.56 (0.09/0.89/0.39)	0.55/0.69/0.36 (0.43/0.5/0.28)
Q31M58	IMP dehydrogenase related 2	0.2/0.29/0.51 (0/0/0)	0.17/0.18/0.26 (0/0/0)	0.31/0.25/0.32 (0/0/0.08)
Q31MS2	Inner membrane protein-like	1.96/2.4/1.84 (0.33/0.36/0.21)	3.16/1.84/1.74 (0.21/0.11/0.52)	2.31/2.99/0.53 (0.28/0.06/0.67)

Q31MF3	Iron-regulated ABC transporter ATPase subunit SufC	4.17/8.17/5.55 (0.26/0/0.2)	1/2.83/4.79 (0.5/0.41/0.09)	8.24/3.5/3.56 (0.04/0.04/0.08)
Q31MY7	Ketol-acid reductoisomerase (NADP(+))	4.83/5.75/4.29 (0/0.04/0.06)	3.98/4.61/3.28 (0/0.06/0.16)	1.05/4.02/2 (0.89/0.08/0.82)
Q31Q43	Membrane-associated 30 kD protein-like	0.06/0.2/0.06 (0/0.03/0)	0.36/0.7/0.49 (0.05/0.57/0)	0.49/0.59/1.25 (0.38/0.02/0.45)
Q31PH4	Methyl-accepting chemotaxis sensory transducer	8.09/2.09/5.15 (0.01/0.99/0.14)	8.55/6.14/13.43 (0/0.11/0.11)	7.11/8.17/12.13 (0.01/0.1/0.1)
Q31PY1	Methyl-accepting chemotaxis sensory transducer with phytochrome sensor	11.59/9.73/10.19 (0.09/0.03/0.01)	17.22/12.13/11.27 (0.05/0/0)	13.18/8.71/5.01 (0.07/0.01/0.22)
Q8GMT0	Nucleoid-associated protein Synpcc7942_0464	6.85/18.54/14.32 (0.1/0/0.21)	10.57/27.8/28.05 (0/0/0.13)	9.04/8.32/20.32 (0.05/0.01/0.17)
P50590	Nucleoside diphosphate kinase	0.2/1.28/0.52 (0.05/0.44/0.44)	0.31/0.33/0.37 (0.17/0.78/0.43)	1.34/1.11/1.45 (0.5/0.67/0.75)
Q07948	ORF1	0.09/0.08/0.49 (0.18/0.02/0.31)	0.2/0.35/0.82 (0.25/0.07/0.56)	0.18/0.09/0.56 (0.24/0.03/0.42)
Q31P53	PBS lyase HEAT-like repeat	0.39/0.31/0.63 (0.22/0/0.29)	0.37/0.16/0.3 (0.29/0/0.1)	0.67/0.52/0.19 (0.45/0.04/0.04)
Q31P88	PDZ/DHR/GLGF	0.33/0.13/0.59 (0.78/0.28/0.33)	0.26/0.15/0.32 (0.66/0.31/0.11)	4.61/1.77/3.08 (0.25/0.36/0.55)
Q31QQ2	PDZ/DHR/GLGF	0.33/0.27/0.9 (0.21/0.09/0.64)	0.63/0.07/0.12 (0.45/0.02/0.06)	1.25/0.83/0.81 (0.65/0.45/0.8)
Q31L49	Peptide methionine sulfoxide reductase MsrB	0.18/0.67/0.13 (0.15/0.05/0)	0.51/0.57/0.15 (0.32/0.07/0.02)	0.7/0.46/0.24 (0.38/0.04/0.12)
Q31N75	Probable porin	0.06/0.3/0.41 (0.08/0.23/0.21)	0.25/0.36/0.33 (0.18/0.17/0.08)	0.31/0.5/0.63 (0.21/0.89/0.51)
Q31N76	Probable porin	0.12/0.16/0.17 (0.17/0.02/0.05)	0.69/0.64/1.13 (0.85/0.71/0.04)	0.25/1.29/1.09 (0.12/0.81/0.94)
P39665	Protein SphX	1.27/4.66/99.08 (0.8/0.39/0.13)	3.91/2.7/99.08 (0.4/0.79/0.4)	2.56/16.29/99.08 (0.45/0.24/0.11)
Q31MT0	Protein splicing (Intein) site	21.68/9.64/2.65 (0.05/0.05/0.01)	18.2/11.8/3.34 (0.06/0.06/0.01)	18.54/9.38/4.66 (0.05/0.14/0)
Q31RZ5	Protein translocase subunit SecD	1.15/1.15/0.78 (0.94/0.65/0.58)	0.34/0.25/0.58 (0.12/0.34/0.39)	1.54/2.05/0.72 (0.38/0.23/0.53)
Q31LC0	RNA methyltransferase TrmH, group 3	0.43/0.47/0.63 (0.28/0.16/0.43)	0.79/0.83/0.4 (0.66/0.52/0.36)	1.49/0.51/0.92 (0.49/0.74/0.84)
Q31Q47	RNA-binding region RNP-1	2.15/14.59/4.21 (0.06/0.02/0.3)	2.05/16.14/4.17 (0.32/0.01/0.26)	5.97/9.04/5.55 (0.03/0.18/0.33)
Q31LP0	RNA-binding region RNP-1	4.97/4.66/4.41 (0.21/0/0.33)	10.28/7.45/16.44 (0.16/0/0.25)	18.03/3.91/13.43 (0.14/0.15/0.24)
Q31RI7	Rod shape-determining protein MreB	0.34/0.49/0.82 (0.07/0.07/0.56)	0.21/0.2/0.28 (0.03/0/0.88)	0.55/0.87/0.95 (0.2/0.35/0.19)
Q31MP0	Rubrerythrin	1.29/0.71/0.49 (0.62/0.42/0.07)	0.77/0.22/0.57 (0.64/0.65/0.08)	0.63/0.4/0.44 (0.45/0.45/0.05)
Q31RK5	Serine hydroxymethyltransferase	4.61/8.39/3.63 (0.02/0.05/0.37)	1.1/2.38/1.84 (0.89/0.14/0.86)	1.41/5.25/4.02 (0.99/0.04/0.26)
Q31RJ2	Sulfate adenylyltransferase	5.11/9.82/4.13 (0.1/0/0)	2.07/3.84/2.83 (0.03/0.28/0.13)	1.57/5.4/1.92 (0.94/0/0.35)
Q31LL7	Transcription termination/antitermination protein NusA	0.5/0.41/1.18 (0.07/0/0.54)	0.25/0.27/0.41 (0/0/0.1)	0.31/0.72/0.59 (0.22/0.02/0.08)
Q31LS0	Transcriptional regulator AbrB	0.69/0.53/0.56 (0.01/0.72/0.83)	0.66/0.4/0.53 (0.02/0.31/0.93)	0.74/0.53/0.17 (0.04/0.32/0.33)

Q935Z3	Trigger factor	2.96/6.43/5.35 (0.17/0.01/0.02)	4.66/3.8/6.14 (0.31/0.18/0.05)	10.38/4.09/7.38 (0.03/0.01/0)
Q31M29	Two component transcriptional regulator, LuxR family	0.44/0.54/0.66 (0.16/0.02/0.42)	0.41/0.98/0.84 (0.12/0.89/0.55)	0.63/0.54/0.71 (0.4/0.34/0.42)
Q31S42	Two component transcriptional regulator, winged helix family	2.29/1.04/1.33 (0.28/0.48/0.81)	5.6/10/2.94 (0.14/0.18/0.28)	2.94/0.87/0.35 (0.22/0.54/0.55)
Q31N86	Two component transcriptional regulator, winged helix family	2.65/8.79/11.17 (0.02/0/0.04)	2.17/7.52/16.6 (0/0/0)	1.2/5.6/5.97 (0.32/0.21/0.07)
Q31QT5	UDP-N-Acetylglucosamine 2-epimerase	0.61/0.82/0.41 (0.78/0.9/0.25)	0.2/0.65/0.43 (0.28/0.25/0.33)	0.38/0.94/0.77 (0.2/0.65/0.47)
Q31MQ1	UPF0367 protein Synpcc7942_1638	0.5/0.58/0.28 (0.58/0.87/0.48)	0.7/1.19/0.43 (0.89/0.83/0.65)	2.7/0.43/0.71 (0.24/0.53/0.86)
Q31QY1	Uridylate kinase	1.01/1.02/0.24 (0.97/0.6/0.22)	0.32/0.36/0.51 (0.21/0.46/0.27)	0.47/2.81/0.12 (0.31/0.29/0.26)

Table S2: Transition list obtained from Skyline for the MRM assay on TSQ Altis™ triple quadrupole mass spectrometer.

Peptide sequence	Start Time (min)	End Time (min)	Precursor (m/z)	Product (m/z)	Collision Energy (V)
DNLELGSDR	12.55	15.55	509.743	789.410108	18.2
DNLELGSDR	12.55	15.55	509.743	676.326044	18.2
DNLELGSDR	12.55	15.55	509.743	547.283451	18.2
DNLELGSDR	12.55	15.55	509.743	434.199387	18.2
DNLELGSDR	12.55	15.55	509.743	343.161211	18.2
DNLELGSDR	12.55	15.55	509.743	472.203804	18.2
SQKPLPMYILAR	19.18	22.18	708.905	1073.617599	24.2
SQKPLPMYILAR	19.18	22.18	708.905	976.564835	24.2
SQKPLPMYILAR	19.18	22.18	708.905	863.480771	24.2
SQKPLPMYILAR	19.18	22.18	708.905	635.387522	24.2
SQKPLPMYILAR	19.18	22.18	708.905	472.324194	24.2
SQKPLPMYILAR	19.18	22.18	708.905	359.24013	24.2
LQGDIDYGDGR	12.99	15.99	604.78	910.390101	21
LQGDIDYGDGR	12.99	15.99	604.78	795.363158	21
LQGDIDYGDGR	12.99	15.99	604.78	682.279094	21
LQGDIDYGDGR	12.99	15.99	604.78	567.252151	21
LQGDIDYGDGR	12.99	15.99	604.78	404.188822	21
LQGDIDYGDGR	12.99	15.99	604.78	347.167359	21
FWSENASFPYK	17.33	20.33	688.319	955.451973	23.6
FWSENASFPYK	17.33	20.33	688.319	826.40938	23.6
FWSENASFPYK	17.33	20.33	688.319	712.366452	23.6
FWSENASFPYK	17.33	20.33	688.319	641.329339	23.6
FWSENASFPYK	17.33	20.33	688.319	554.29731	23.6
FWSENASFPYK	17.33	20.33	688.319	407.228896	23.6
WGYLPASTDTK	15.98	18.98	619.806	832.441074	21.5
WGYLPASTDTK	15.98	18.98	619.806	719.35701	21.5
WGYLPASTDTK	15.98	18.98	619.806	622.304246	21.5

WGYLPASTDTK	15.98	18.98	619.806	551.267132	21.5
WGYLPASTDTK	15.98	18.98	619.806	464.235104	21.5
WGYLPASTDTK	15.98	18.98	619.806	363.187425	21.5
MNQVEMLGR	14.22	17.22	539.262	832.434549	19.1
MNQVEMLGR	14.22	17.22	539.262	704.375971	19.1
MNQVEMLGR	14.22	17.22	539.262	605.307557	19.1
MNQVEMLGR	14.22	17.22	539.262	476.264964	19.1
MNQVEMLGR	14.22	17.22	539.262	345.22448	19.1
MNQVEMLGR	14.22	17.22	539.262	232.140415	19.1
MNQVEMLGR	14.22	17.22	539.262	374.149266	19.1
GGLFAAPDFYMK	20.72	23.72	658.82	1089.507379	22.7
GGLFAAPDFYMK	20.72	23.72	658.82	942.438965	22.7
GGLFAAPDFYMK	20.72	23.72	658.82	871.401852	22.7
GGLFAAPDFYMK	20.72	23.72	658.82	800.364738	22.7
GGLFAAPDFYMK	20.72	23.72	658.82	703.311974	22.7
GGLFAAPDFYMK	20.72	23.72	658.82	588.285031	22.7
GGLFAAPDFYMK	20.72	23.72	658.82	441.216617	22.7
TQSLVISSQSR	12.1	15.1	603.327	889.510157	21
TQSLVISSQSR	12.1	15.1	603.327	776.426093	21
TQSLVISSQSR	12.1	15.1	603.327	677.357679	21
TQSLVISSQSR	12.1	15.1	603.327	564.273615	21
TQSLVISSQSR	12.1	15.1	603.327	477.241586	21
TQSLVISSQSR	12.1	15.1	603.327	390.209558	21
SLLNQGYR	13.12	16.12	475.756	750.389313	17.2
SLLNQGYR	13.12	16.12	475.756	637.305249	17.2
SLLNQGYR	13.12	16.12	475.756	523.262322	17.2
SLLNQGYR	13.12	16.12	475.756	395.203744	17.2
SLLNQGYR	13.12	16.12	475.756	338.18228	17.2
SLLNQGYR	13.12	16.12	475.756	314.207432	17.2
SLLNQGYR	13.12	16.12	475.756	428.25036	17.2
LIGIDPAAR	15.07	18.07	463.276	699.378414	16.8
LIGIDPAAR	15.07	18.07	463.276	642.35695	16.8
LIGIDPAAR	15.07	18.07	463.276	529.272886	16.8
LIGIDPAAR	15.07	18.07	463.276	414.245943	16.8
LIGIDPAAR	15.07	18.07	463.276	284.196868	16.8
LIGIDPAAR	15.07	18.07	463.276	397.280932	16.8
VAELLIQKP	15.56	18.56	505.815	840.51893	18.1
VAELLIQKP	15.56	18.56	505.815	711.476337	18.1
VAELLIQKP	15.56	18.56	505.815	598.392273	18.1
VAELLIQKP	15.56	18.56	505.815	485.308209	18.1
VAELLIQKP	15.56	18.56	505.815	372.224145	18.1
VAELLIQKP	15.56	18.56	505.815	300.155397	18.1
VAELLIQKP	15.56	18.56	505.815	413.239461	18.1
SPQQVLDEVR	14.88	17.88	585.809	858.467957	20.5
SPQQVLDEVR	14.88	17.88	585.809	730.40938	20.5
SPQQVLDEVR	14.88	17.88	585.809	631.340966	20.5
SPQQVLDEVR	14.88	17.88	585.809	518.256902	20.5
SPQQVLDEVR	14.88	17.88	585.809	403.229959	20.5
SEYGDC[+57.021464]YIR	13.67	16.67	581.745	946.408728	20.4
SEYGDC[+57.021464]YIR	13.67	16.67	581.745	783.3454	20.4

SEYGDC[+57.021464]YIR	13.67	16.67	581.745	726.323936	20.4
SEYGDC[+57.021464]YIR	13.67	16.67	581.745	611.296993	20.4
SEYGDC[+57.021464]YIR	13.67	16.67	581.745	451.266344	20.4
SEYGDC[+57.021464]YIR	13.67	16.67	581.745	288.203016	20.4
VAGFDNIK	13.48	16.48	432.234	764.39373	15.9
VAGFDNIK	13.48	16.48	432.234	693.356616	15.9
VAGFDNIK	13.48	16.48	432.234	636.335152	15.9
VAGFDNIK	13.48	16.48	432.234	489.266738	15.9
VAGFDNIK	13.48	16.48	432.234	374.239795	15.9
VAGFDNIK	13.48	16.48	432.234	260.196868	15.9
SPTGEIIFGGETMR	17.2	20.2	747.866	1209.593234	25.3
SPTGEIIFGGETMR	17.2	20.2	747.866	1152.571771	25.3
SPTGEIIFGGETMR	17.2	20.2	747.866	1023.529177	25.3
SPTGEIIFGGETMR	17.2	20.2	747.866	910.445113	25.3
SPTGEIIFGGETMR	17.2	20.2	747.866	797.361049	25.3
SPTGEIIFGGETMR	17.2	20.2	747.866	650.292636	25.3
SPTGEIIFGGETMR	17.2	20.2	747.866	593.271172	25.3
SPTGEIIFGGETMR	17.2	20.2	747.866	407.207115	25.3
GPWLEPLR	16.92	19.92	484.271	813.46175	17.4
GPWLEPLR	16.92	19.92	484.271	627.382437	17.4
GPWLEPLR	16.92	19.92	484.271	514.298373	17.4
GPWLEPLR	16.92	19.92	484.271	385.25578	17.4
GPWLEPLR	16.92	19.92	484.271	341.160817	17.4
GPWLEPLR	16.92	19.92	484.271	454.244881	17.4
LTNDIQPWQAR	15.33	18.33	671.349	1013.516305	23
LTNDIQPWQAR	15.33	18.33	671.349	898.489362	23
LTNDIQPWQAR	15.33	18.33	671.349	785.405297	23
LTNDIQPWQAR	15.33	18.33	671.349	657.34672	23
LTNDIQPWQAR	15.33	18.33	671.349	374.214643	23
GIDILAEAVAVTLGPK	27.27	30	783.958	1055.609536	26.4
GIDILAEAVAVTLGPK	27.27	30	783.958	984.572423	26.4
GIDILAEAVAVTLGPK	27.27	30	783.958	855.529829	26.4
GIDILAEAVAVTLGPK	27.27	30	783.958	784.492716	26.4
GIDILAEAVAVTLGPK	27.27	30	783.958	685.424302	26.4
GIDILAEAVAVTLGPK	27.27	30	783.958	301.187031	26.4
GYISPYFATDTER	16.92	19.92	760.356	1186.537494	25.7
GYISPYFATDTER	16.92	19.92	760.356	1099.505465	25.7
GYISPYFATDTER	16.92	19.92	760.356	1002.452701	25.7
GYISPYFATDTER	16.92	19.92	760.356	839.389373	25.7
GYISPYFATDTER	16.92	19.92	760.356	692.320959	25.7
GYISPYFATDTER	16.92	19.92	760.356	405.209223	25.7
EALATLVVNR	15.76	18.76	543.319	772.467563	19.2
EALATLVVNR	15.76	18.76	543.319	701.43045	19.2
EALATLVVNR	15.76	18.76	543.319	600.382771	19.2
EALATLVVNR	15.76	18.76	543.319	487.298707	19.2
EALATLVVNR	15.76	18.76	543.319	388.230293	19.2
DNTTIVAEGNEAAVK	13.98	16.98	766.383	1100.594615	25.9
DNTTIVAEGNEAAVK	13.98	16.98	766.383	987.510551	25.9
DNTTIVAEGNEAAVK	13.98	16.98	766.383	888.442137	25.9
DNTTIVAEGNEAAVK	13.98	16.98	766.383	817.405023	25.9

DNTTIVAEGNEAAVK	13.98	16.98	766.383	688.36243	25.9
DNTTIVAEGNEAAVK	13.98	16.98	766.383	388.255445	25.9
QQIEQYSQDVK	12.46	15.46	683.335	996.463266	23.4
QQIEQYSQDVK	12.46	15.46	683.335	867.420673	23.4
QQIEQYSQDVK	12.46	15.46	683.335	739.362095	23.4
QQIEQYSQDVK	12.46	15.46	683.335	576.298767	23.4
QQIEQYSQDVK	12.46	15.46	683.335	489.266738	23.4
QQIEQYSQDVK	12.46	15.46	683.335	361.208161	23.4
VENVGTVLQVGDGIAR	17.45	20.45	813.944	1185.658612	27.3
VENVGTVLQVGDGIAR	17.45	20.45	813.944	1128.637148	27.3
VENVGTVLQVGDGIAR	17.45	20.45	813.944	1027.58947	27.3
VENVGTVLQVGDGIAR	17.45	20.45	813.944	928.521056	27.3
VENVGTVLQVGDGIAR	17.45	20.45	813.944	815.436992	27.3
VENVGTVLQVGDGIAR	17.45	20.45	813.944	687.378414	27.3
VENVGTVLQVGDGIAR	17.45	20.45	813.944	588.31	27.3
VENVGTVLQVGDGIAR	17.45	20.45	813.944	416.261593	27.3
IAQIPVGDALVGR	17.08	20.08	654.885	996.583656	22.6
IAQIPVGDALVGR	17.08	20.08	654.885	883.499592	22.6
IAQIPVGDALVGR	17.08	20.08	654.885	786.446828	22.6
IAQIPVGDALVGR	17.08	20.08	654.885	687.378414	22.6
IAQIPVGDALVGR	17.08	20.08	654.885	515.330007	22.6
IAQIPVGDALVGR	17.08	20.08	654.885	331.208829	22.6
VVSPLGAPLDGK	15.64	18.64	576.834	867.493444	20.2
VVSPLGAPLDGK	15.64	18.64	576.834	770.44068	20.2
VVSPLGAPLDGK	15.64	18.64	576.834	657.356616	20.2
VVSPLGAPLDGK	15.64	18.64	576.834	600.335152	20.2
VVSPLGAPLDGK	15.64	18.64	576.834	529.298038	20.2
VVSPLGAPLDGK	15.64	18.64	576.834	319.161211	20.2
IVPQELVR	14.42	17.42	477.292	741.425364	17.2
IVPQELVR	14.42	17.42	477.292	644.3726	17.2
IVPQELVR	14.42	17.42	477.292	516.314023	17.2
DTNPTEGVNC[+57.021464]ITK	21.21	24.21	724.837	635.318122	24.7
DTNPTEGVNC[+57.021464]ITK	21.21	24.21	724.837	521.275195	24.7
DTNPTEGVNC[+57.021464]ITK	21.21	24.21	724.837	361.244546	24.7
GVSDEFVEPTR	14.73	17.73	618.298	992.468351	21.5
GVSDEFVEPTR	14.73	17.73	618.298	877.441408	21.5
GVSDEFVEPTR	14.73	17.73	618.298	748.398815	21.5
GVSDEFVEPTR	14.73	17.73	618.298	601.330401	21.5
GVSDEFVEPTR	14.73	17.73	618.298	502.261987	21.5
GVSDEFVEPTR	14.73	17.73	618.298	373.219394	21.5
MGATVEAIETVDR	20.69	23.69	696.345	932.468351	23.8
MGATVEAIETVDR	20.69	23.69	696.345	732.388644	23.8
MGATVEAIETVDR	20.69	23.69	696.345	490.261987	23.8
MGATVEAIETVDR	20.69	23.69	696.345	389.214309	23.8
MGATVEAIETVDR	20.69	23.69	696.345	361.154017	23.8
MGATVEAIETVDR	20.69	23.69	696.345	460.222431	23.8
AGIETYLIER	17.19	20.19	582.816	923.483273	20.4
AGIETYLIER	17.19	20.19	582.816	794.44068	20.4
AGIETYLIER	17.19	20.19	582.816	693.393001	20.4
AGIETYLIER	17.19	20.19	582.816	530.329673	20.4

AGIETYLIER	17.19	20.19	582.816	417.245609	20.4
AGIETYLIER	17.19	20.19	582.816	304.161545	20.4
AALVGDAAGYVTK	15.2	18.2	618.335	980.504737	21.5
AALVGDAAGYVTK	15.2	18.2	618.335	881.436323	21.5
AALVGDAAGYVTK	15.2	18.2	618.335	824.414859	21.5
AALVGDAAGYVTK	15.2	18.2	618.335	709.387916	21.5
AALVGDAAGYVTK	15.2	18.2	618.335	638.350802	21.5
AALVGDAAGYVTK	15.2	18.2	618.335	567.313689	21.5
AALVGDAAGYVTK	15.2	18.2	618.335	347.228896	21.5
SSGEGIYFAAK	14.42	17.42	565.279	898.466895	19.9
SSGEGIYFAAK	14.42	17.42	565.279	769.424302	19.9
SSGEGIYFAAK	14.42	17.42	565.279	712.402838	19.9
SSGEGIYFAAK	14.42	17.42	565.279	599.318774	19.9
SSGEGIYFAAK	14.42	17.42	565.279	436.255445	19.9
SSGEGIYFAAK	14.42	17.42	565.279	289.187031	19.9
VLDLLQTVFYR	24.43	27.43	683.89	1039.593492	23.4
VLDLLQTVFYR	24.43	27.43	683.89	926.509428	23.4
VLDLLQTVFYR	24.43	27.43	683.89	813.425364	23.4
VLDLLQTVFYR	24.43	27.43	683.89	685.366787	23.4
VLDLLQTVFYR	24.43	27.43	683.89	584.319108	23.4
VLDLLQTVFYR	24.43	27.43	683.89	485.250694	23.4
TVVPANPLVQLK	17.2	20.2	639.892	979.593492	22.1
TVVPANPLVQLK	17.2	20.2	639.892	882.540728	22.1
TVVPANPLVQLK	17.2	20.2	639.892	811.503615	22.1
TVVPANPLVQLK	17.2	20.2	639.892	697.460687	22.1
TVVPANPLVQLK	17.2	20.2	639.892	388.255445	22.1
DGTPGC[+57.021464]TLEAQR	11.98	14.98	652.798	1031.493855	22.5
DGTPGC[+57.021464]TLEAQR	11.98	14.98	652.798	934.441091	22.5
DGTPGC[+57.021464]TLEAQR	11.98	14.98	652.798	717.388979	22.5
DGTPGC[+57.021464]TLEAQR	11.98	14.98	652.798	616.3413	22.5
DGTPGC[+57.021464]TLEAQR	11.98	14.98	652.798	503.257236	22.5
DGTPGC[+57.021464]TLEAQR	11.98	14.98	652.798	374.214643	22.5
YGSWLAPFSLR	22.59	25.59	648.84	989.556713	22.4
YGSWLAPFSLR	22.59	25.59	648.84	803.4774	22.4
YGSWLAPFSLR	22.59	25.59	648.84	690.393336	22.4
YGSWLAPFSLR	22.59	25.59	648.84	619.356222	22.4
HTYIIDPEGVLR	19.61	22.61	706.88	1011.583322	24.1
HTYIIDPEGVLR	19.61	22.61	706.88	898.499258	24.1
HTYIIDPEGVLR	19.61	22.61	706.88	670.38825	24.1
HTYIIDPEGVLR	19.61	22.61	706.88	444.292893	24.1
